# Molecular classification and therapeutics in diffuse large B-cell lymphoma

**DOI:** 10.3389/fmolb.2023.1124360

**Published:** 2023-02-03

**Authors:** Gaelen Shimkus, Taichiro Nonaka

**Affiliations:** ^1^ School of Medicine, Louisiana State University Health Shreveport, Shreveport, LA, United States; ^2^ Department of Cellular Biology and Anatomy, Louisiana State University Health Sciences Center, Shreveport, LA, United States; ^3^ Feist-Weiller Cancer Center, Louisiana State University Health Shreveport, Shreveport, LA, United States

**Keywords:** DLBCL, molecular diagnostics, molecular classification, signaling pathway, targeted therapy

## Abstract

Diffuse large B-cell lymphoma (DLBCL) encompasses a wide variety of disease states that have to date been subgrouped and characterized based on immunohistochemical methods, which provide limited prognostic value to clinicians and no alteration in treatment regimen. The addition of rituximab to CHOP therapy was the last leap forward in terms of treatment, but regimens currently follow a standardized course when disease becomes refractory with no individualization based on genotype. Research groups are tentatively proposing new strategies for categorizing DLBCL based on genetic abnormalities that are frequently found together to better predict disease course following dysregulation of specific pathways and to deliver targeted treatment. Novel algorithms in combination with next-generation sequencing techniques have identified between 4 and 7 subgroups of DLBCL, depending on the research team, with potentially significant and actionable genetic alterations. Various drugs aimed at pathways including BCR signaling, NF-κB dysfunction, and epigenetic regulation have shown promise in their respective groups and may show initial utility as second or third line therapies to patients with recurrent DLBCL. Implementation of subgroups will allow collection of necessary data to determine which groups are significant, which treatments may be indicated, and will provide better insight to clinicians and patients on specific disease course.

## 1 Introduction

Diffuse large B-cell lymphoma (referred to as DLBCL) is characterized by a consistent range of cellular precursors and nuclear morphology, namely large B lymphoid cells with nuclei exceeding normal lymphocyte size, but spans many immunologic and genetic subtypes without much further classification (Stein et al., 2008). Immunohistochemistry in combination with various algorithms (which can differ from lab to lab) have allowed for basic classification of DLBCL into two groups, activated B-cell like type (ABC) and germinal center B-cell like type (GCB) which has provided prognostic insight into disease course without yielding much into targeted therapies for these distinct subtypes (Cerami et al., 2012; Tilly et al., 2015). In addition, this stratification system is largely a phenotypic description of DLBCL as opposed to a genetic description, so treatment responses are not fully explained by these subtypes (Wright et al., 2020). Clinically relevant stratification of DLBCL is largely non-existent and a classification such as a combination of CD10, BCL6, and IRF4/MUM1 expression, so-called “Hans classifier” was proposed to be useful in predicting long or short term survival (Hans et al., 2004).

The International Consensus Classification of Mature Lymphoid Neoplasms, which aims to provide standardized diagnostic criteria for lymphoid malignancies to pathologists, geneticists, scientists, and clinicians recognizes the utility of cell-of-origin (COO) designation in DLBCL, but also acknowledges that the system has shortcomings (Campo et al., 2022). Stratification by purely COO studies is considered insufficient to fully capture the genetic diversity of these tumors, especially relating to patient outcomes and treatment options, and essentially represents only the end result of faulty or mutated genetic pathways. Further classification may be warranted for DLBCL occurring in extranodal locations or immune privileged sites such as the central nervous system or testis. Inclusion of this extranodal branch of DLBCL in future classification systems may be warranted because these malignancies tend to share similar genetic alterations, such as a high prevalence of *MYD88* ^L265P^ and *CD79B* mutations, which are also defining characteristics of MCD/C5 genetic subgroups, which are to be expanded upon further in this review.

The fifth edition of the World Health Organization Classification of Haematolymphoid Tumours: Lymphoid Neoplasms (WHO-HAEM5) exists to provide global definitions and classification systems for lymphoid tumors, and is intended for use by pathologists, clinicians, and research scientists (Alaggio et al., 2022). Characterization of DLBCL, otherwise known in the referenced paper as “Large B-cell lymphomas”, are defined by morphology and must be carefully and meticulously differentiated from other malignancies such as blastoid variant of mantle cell lymphoma or lymphoblastic lymphoma, which can appear similar. In this globally accepted system for the identification and diagnosis of DLBCL the heterogeneity of DLBCL is greatly emphasized, and the recommendation for further rendering of ABC/GCB subtypes is made due to the lack impact that this system has on clinical outcomes for patients. DLBCL that does not fall into ABC or GCB categories is listed as DLBCL NOS (DLBCL not otherwise specified) and this alone has over 150 identified genetic drivers that occur in various combinations that ultimately lead to neoplasia and disease. The utility of next-generation sequencing (NGS) with novel clustering algorithms is acknowledged, and patterns have begun to emerge such as the similarity of genetic subtypes to follicular lymphoma or mantle cell lymphoma, which may also imply an overlap in possible treatment, and may suggest the possibility of genetic founder effects that initiate the cascade of mutations. WHO-HAEM5 overall emphasizes the importance of new subtypes for diagnosis and treatment, but awaits data from clinical trials before attempting to publish novel diagnostic subclasses of DLBCL.

National Comprehensive Cancer Network (NCCN) guidelines direct practitioners to standardized R-CHOP therapy (rituximab, cyclophosphamide, doxorubicin hydrochloride, vinicristine sulfate, and prednisone) as first line treatment regardless of presentation or cellular markers, and refractory cases are treated uniformly with further R-CHOP therapy at higher dosage, second line therapy in combination with hematopoietic cell transplant, chimeric antigen receptor T-cell (CAR T-cell) therapy, clinical trials, or finally palliative care (Tilly et al., 2015). The second line therapies are dependent on whether or not the patient and clinician intend on proceeding to hematopoietic stem cell transplant, as well as other confounding factors such as presence of poor left ventricular function. The introduction of rituximab, a chimeric anti-CD20 monoclonal antibody, was the most recent and last leap forward in treatment of DLBCL, and was added to previous first line therapies to create the newest standard of care treatment R-CHOP (Coiffier, 2007). Trials conducted using regimens other than R-CHOP have failed to demonstrate significant advantage over the current first line therapy (Coiffier, 2007).

Identification of genetic markers that alter disease course is essential not only for implementation of effective treatment, but also to indicate which patients may require less potent treatment or less invasive surveillance in looking for relapse. New therapies derived from NGS provided data are aimed at disrupting signaling pathways or modulating immune response, however the impact of these therapies can only be fully realized when we are able to categorize DLBCL into subtypes based on internal disease mechanism, as opposed to outward morphology or presence of basic cell markers. These internal mechanisms, and sorting the influential from the irrelevant, are the basis for future treatment modalities and the cessation of indiscriminate therapy based on patient response. In this paper we will begin by exploring the various diagnostic criteria for subtyping of DLBCL on a molecular level, and the genetic alterations that change basic signaling pathways and cascades that determine disease progression and outcome. Targeted therapies aimed at these pathways will be investigated, and lastly the clinical implications and research still needed will be discussed.

## 2 Molecular diagnosis and classification of DLBCL

DLBCL can currently be divided into 3 subtypes based on cell of origin (COO), ABC, GCB, and Unclassified, which yield moderate prognostic value and decidedly limited clinical value due to the standard progression of treatment regimens across all three types (Schmitz et al., 2018). These subtypes are based primarily on which stage in B cell development these malignant cells most resemble morphologically and genetically (ABC/GCB), or the absence of identifying characteristics (Unclassified) which is the broadest subtype (Campo et al., 2011; Rosenquist et al., 2016; Rosenquist et al., 2017). While these subtypes do share high incidences of similar genetic mutations within their respective group (e.g., B-cell receptor or NOTCH abnormalities), these groups are insufficient for the implementation of precision treatment and prediction of disease course, especially in disease that is non-responsive to traditional R-CHOP therapy. Heterozygosity among DLBCL within ABC/GCB/Unclassified groups is the primary barrier to treatment with a uniform regimen like R-CHOP and explains the need for a narrower and more well-defined set of groups with targeted treatment options.

**FIGURE 1:**
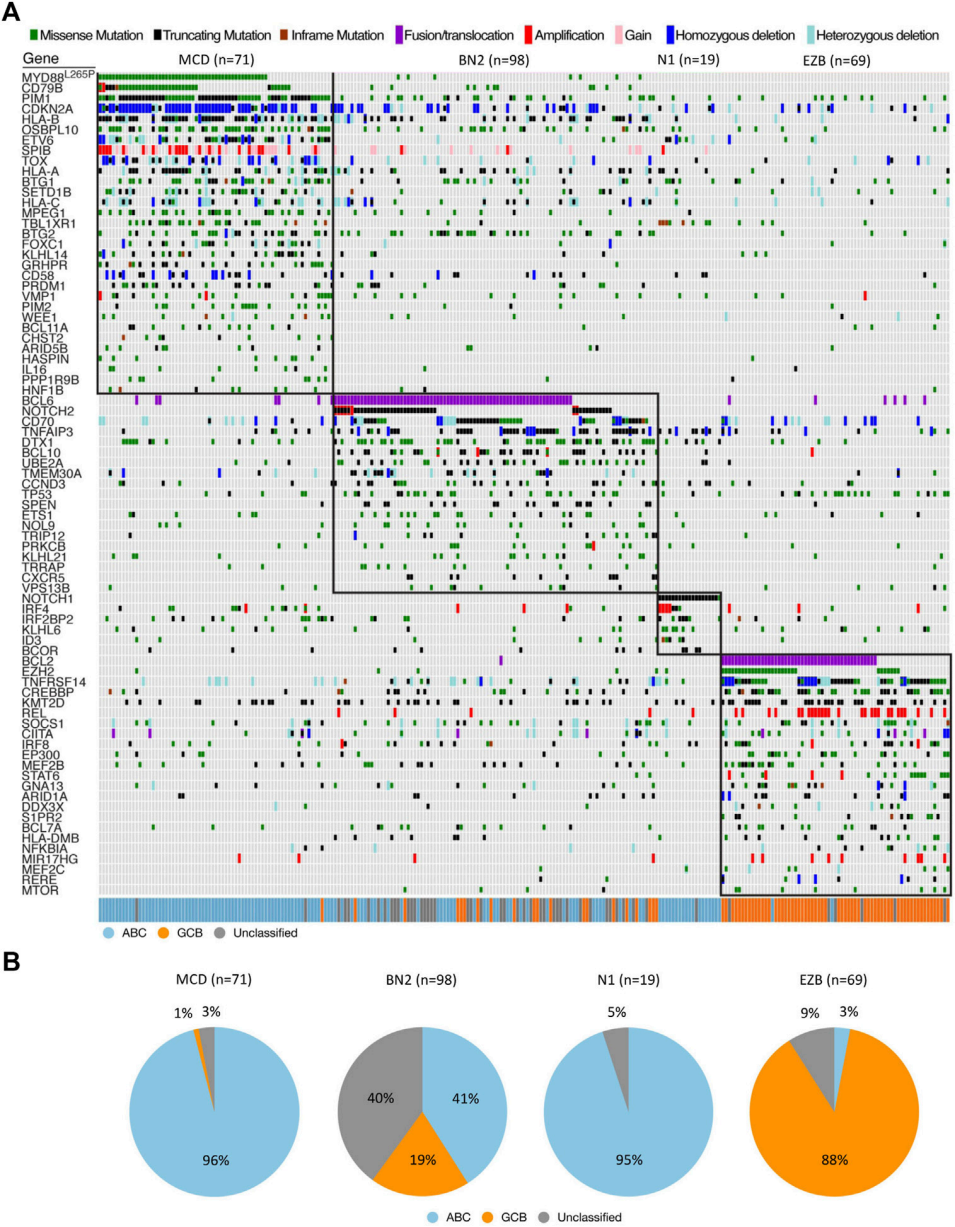
Genetic features of DLBCL. **(A)** Landscape of DLBCL genetic subtypes based on genetic alteration. Relationship between genetic subtypes (e.g., MCD, BN2, N1, EZB) and gene expression subtypes (e.g., ABC, GCB, Unclassified) is also shown. **(B)** Distribution of gene expression subtypes within genetic subtypes. All data were extracted from the results of the whole exome sequence and RNA-seq published by Schmitz et al. (2018). Adapted with permission from Schmitz et al. (2018). Copyright 2018, Massachusetts Medical Society.

NGS has allowed for the analysis of extremely large DLBCL samples in order to differentiate impactful pathogenetic sequences from those that do not significantly alter disease course, as well as provide a meaningful way of typing cells more accurately than the current standard (ABC/GCB grouping based off variable immunochemistry) (Mansouri et al., 2022). A study conducted by Schmitz et al. subdivided DLBCL into 4 groups (EZB, BN2, MCD, N1) based on co-occurrence of predefined sequences and mutations in an effort to look at mutations as a “constellation” of abnormalities that defines the subtype and thus forms the specific disease state (Figure 1A) (Schmitz et al., 2018). These four subtypes were demonstrated to have distinctly different progression-free survival (PFS) and overall survival (OS) associated with them, as well as more predictable disease course such as trends towards extra-nodal involvement as seen in the MCD subtype (Puente et al., 2015; Schmitz et al., 2018). These delineations are meaningful not only prognostically, but differing response to treatment has been observed among the groups, such as the increased susceptibility of the MCD subtype to BCR inhibitors (Wright et al., 2020).

### 2.1 Subgroup by cell of origin (COO)

ABC, GCB, and Unclassified DLBCL are divided based on stage of development they most resemble after some genetic mutation disrupts typical progression and causes malignant transformation. The cells of these subtypes develop their morphologic and clinical characteristics based on the stage of development in which they accumulate enough genetic variation to become malignant, and differentiation either ceases or continues on a path not seen by healthy and functional B cells (Schmitz et al., 2018). The ABC subtype is thought to have progressed through the germinal center and is committed to plasmablastic differentiation, while GCB cells are thought to originate from light zones in the germinal center (Pfreundschuh et al., 2008). Clinical outcomes of GCB lymphomas are widely recognized as superior to ABC malignancies when traditional R-CHOP therapy is utilized, however the risk stratification has not led to improvements in treatment outcome. 30%–50% of patients with DLBCL will not respond to this therapy, and only 10% with disease refractory to this treatment will be cured with second line salvage treatment or bone marrow transplants (Feugier et al., 2005; Pfreundschuh et al., 2006; Pfreundschuh et al., 2008; Gisselbrecht et al., 2010; Friedberg, 2011; Coiffier and Sarkozy, 2016). The heterogeneity of treatment outcomes, even among ABC, GCB, and Unclassified subtypes likely results from the specific pathways with altered regulation, expression, or end products that are not defined by classical subtyping.

#### 2.1.1 Activated B-cell like type (ABC)

Cells currently falling under the ABC classification to tend to express common mutations and translocations, such as *PRDM1* truncations or homozygous deletions only seen in the ABC subtype, that have given insight into their behavior patterns (Pasqualucci et al., 2006; Mandelbaum et al., 2010; Schmitz et al., 2018). Identification of these defining pathways and association with a specific disease course, albeit in mice, further backs the strength of disease grouping in prognostics and treatment options (Mandelbaum et al., 2010; Miao et al., 2019). These cells show increased incidence of “chronic active” BCR signaling which is characterized by BCR clustering and autoreactive self-antigens as opposed to tonic signaling which is antigen independent and exhibits a lack of BCR clustering, as seen in GCB. *MYD88* mutations conferring extranodal involvement, TNFAIP3 inactivation leading to uncontrollable NF-κB expression, and *NOTCH1* mutations are seen almost exclusively in this subtype. These mutations however are not seen in a majority of cells expressing the ABC phenotype so they can hardly be called ABC defining traits, but are far more strongly associated with this subtype than the GCB or Unclassified types.

#### 2.1.2 Germinal center B-cell like type (GCB)

GCB cells are affected by the master regulator BCL6 similarly to ABC cells, but also are affected by more unique mutations. These possess an association with *REL* amplifications promoting lymphomagenesis, an almost exclusive presentation of t(14;18)(q32;q21) translocations leading to BCL2 activation and overexpression, and *CREBBP* mutation affecting the histone acetyltransferase domain leading to epigenetic dysregulation (Iqbal et al., 2004; Kusumoto et al., 2005; Kridel et al., 2012; Visco et al., 2013; Ennishi et al., 2017; Miao et al., 2019). The incidence of these mutations among all patients diagnosed with GCB DLBCL is relatively low and again can hardly be called defining to the disease type. The presence of these mutations in a GCB DLBCL and the association of GCB type with superior response to first line R-CHOP therapy supports the somewhat obvious notion that different (dysregulated) pathways respond differently, or not at all, to a given treatment.

One of the biggest problems with the accepted stratification of DLBCL is the disparity in treatment response even in the presence of seemingly identical focal mutations in both ABC and GCB, such as *TP53* deletion or *MYC* mutations (Jia et al., 2012; Xu-Monette et al., 2012; Cao et al., 2016; Xu-Monette et al., 2016). This observation coupled with the vast number of possible mutations suggests not that we need groups based on individual pathway alterations, but the need for grouping based on sets of mutations that can be found together and that can be targeted therapeutically (Mansouri et al., 2022).

### 2.2 Subgroup by genetic alteration and signaling pathway

The current ABC and GCB subgroups provide relatively little utility because they do not take into account the numerous changes that can occur within the genome that are not observable with immunohistochemical staining techniques. In order to develop targeted treatments and better predict disease course, we must take a closer look at which pathways specifically are causing the dysplastic growth of these cells, where these pathways have gone awry, and if they are even impactful to the overall progression of the malignancy. We will now identify the pathways that have been deemed influential by various research teams, and that help define the proposed subgroups (Table 1).

**TABLE 1:** Molecular classification of DLBCL. *Prevalance data were extracted from the results published by Wright et al. (2020).

Molecular classification				
Wright	Schmitz	Lacy	Chapuy	Genetic alteration (% prevalance*)
BN2	BN2	NOTCH2	C1	*BCL6* (72.8%), *NOTCH2* (41.8%), *TNFAIP3* (51.6%), *DTX1* (50.0%), *CD70* (41.3%), *BCL10* (39.6%), *UBE2A* (30.4%), *TMEM30A* (26.7%), *KLF2* (21.7%), *SPEN* (21.7%)
A53	—	—	C2	*TP53* (86.8%), *B2M* (34.2), *TP53BP1* (27.0%), *CNPY3* (23.7%), *ING1* (15.8%), *NFKBIZ* (15.8%), *TP73* (13.2%)
EZB-MYC+ EZB-MYC–	EZB	BCL2	C3	*BCL2* (68.4%), *EZH2* (44.7%), *TNFRSF14* (66.2%), *KMT2D* (53.9%), *CREBBP* (52.7%), *REL* (34.3%), *FAS* (30.1%), *IRF8* (28.9%), *EP300* (27.8%), *MEF2B* (26.3%), *CIITA* (25.0%), *ARID1A* (22.9%), *GNA13* (22.5%), *STAT6* (21.1%), *PTEN* (20.0%)
ST2	—	TET2/SGK1SOCS1/SGK1	C4	*TET2* (48.1%), *DUSP2* (44.4%), *ZFP36L1* (40.7%), *ACTG1* (37.0%), *SGK1* (37.0%), *ITPKB* (33.3%), *NFKBIA* (33.3%), *EIF4A2* (29.6%), *JUNB* (29.6%), *STAT3* (29.6%), *BCL2L1* (25.9%), *CD83* (25.9%), *DDX3X* (25.9%), *SOCS1* (25.9%), *CD83* (25.9%), *P2RY8* (22.2%), *RFTN1* (22.2%)
MCD	MCD	MYD88	C5	*MYD88* (66.2%), *CD79B* (50.0%), *PIM1* (92.5%), *HLA-B* (73.8%), *BTG1* (70.0%), *CDKN2A* (62.0%), *ETV6* (55.0%), *SPIB* (51.9%), *OSBPL10* (51.2%), *TOX* (48.1%), *BCL2* (48.1%), *BTG2* (43.8%), *MPEG1* (43.8%), *HLA-A* (43.0%), *HLA-C* (42.5%), *SETD1B* (41.8%), *KLHL14* (41.2%), *TBL1XR1* (35.0%), *GRHPR* (33.8%), *PRDM1* (32.5%), *CD58* (31.6%), *TAP1* (26.6%), *PIM2* (25.0%), *FOXC1* (21.2%), *IRF4* (20.0%)
N1	N1	—	—	*NOTCH1* (100%), *IRF2BP2* (43.8%), *ID3* (25.0%), *BCOR* (25.0%), *EPB41* (18.8%), *IKBKB* (18.8%), *ALDH18A1* (18.8%)

#### 2.2.1 Schmitz’s classification: BN2, EZB, MCD, N1

A classification system developed by Schmitz et al. (2018) has sought to link certain groupings of mutations with disease course, severity, and response to traditional R-CHOP therapy as well as suggest other potential therapies. Their view of genetic anomalies was not to view each mutation as an independent and unrelated event, but to try and group mutations that commonly occurred together and characterized a distinct disease course. Independent mutations would be nearly impossible to keep track of and treat individually at this stage in our knowledge and practice of medicine, so groupings like these encompassing multiple class defining mutations could prove to be actionable. The four groups of DLBCL they established through use of their algorithm were “MCD” defined by *MYD88* and *CD79B* mutations, “BN2” defined by *BCL6* fusions and *NOTCH2* mutations, “N1” defined by *NOTCH1* mutations, and “EZB” defined by *EZH2* and *BCL2* translocations. ABC cells in the MCD category were observed to have a superior response to ibrutinib, which halts B-cell proliferation through action as a Bruton’s tyrosine kinase (BTK) inhibitor, supporting the notion that better characterization of genetic phenomena and heterogeneity can lead to more effective and targeted treatment (Shaffer et al., 2006; Puente et al., 2015; Chapuy et al., 2016). Mutations found more commonly in ABC or GCB subtypes respectively further add defining features and explanation to their course, such as N1 subtype appearing in ABC 95% of the time, or the observed co-occurrence of *EZH2* mutations with *BCL2* translocations in GCB type lymphomas (Figure 1B) (Miao et al., 2019). The newly defined subtypes also observed significant progression-free survival (PFS) differences, with best outcomes going to BN2 and EZB types (Schmitz et al., 2018). Interestingly these subtypes, BN2 and EZB, were most commonly seen in the current GCB classification which is already accepted as having superior response and survival rate to ABC type malignancies (Cerami et al., 2012; Gao et al., 2013; Valls et al., 2017; Schmitz et al., 2018). The alignment between the potential new classification types and the currently accepted system adds a layer of confidence in exploring the subtypes proposed by Schmitz et al. in terms of prognostic indication, and development of treatments targeted at the pathways identified in their algorithm.

#### 2.2.2 Chapuy’s classification: C1-C5

Chapuy et al. have developed another classification system through use of whole exome sequencing (WES) that divides DLBCL cells into C1, C2, C3, C4, or C5 depending on pattern of gene expression with defining genetic drivers, or C0 if a genetic driver could not be identified (Chapuy et al., 2018). Cluster 5 exhibited a consistent 18q gain, and is notable because 8 of the 9 patients in the Chapuy sample that had testicular involvement fell into the C5 cluster and 1 of 2 patients in the sample with CNS involvement fell into this cluster (Wright et al., 2003; Monti et al., 2005). This correlation is exciting for two reasons, first it shows a relation between disease course and genetic drivers in DLBCL which has the potential to aid in targeted screening for patients with identified mutations (e.g., 18q deletion) and in selecting more appropriate therapies. Secondarily the C5 group displaying increased extranodal involvement is significant because genes observed in the C5 group (e.g., *MYD88*) overlap heavily with the genes observed in the Schmitz et al. subgroup MCD, where extranodal involvement was also noted to be significant (Table 1). While the grouping of genes is slightly different between MCD and C5, the overlap and mutually identified association with specific disease course emphasizes the utility of being able to subtype DLBCL beyond what is currently used. Subtype C1 shared alterations in certain pathways (e.g., NOTCH2) with low grade marginal zones lymphomas, and alterations in other NOTCH2 and BCL6 pathways were defining characteristics of this group (Li and Durbin, 2009; Chapuy et al., 2013; Scott et al., 2014; Chapuy et al., 2016; Staiger et al., 2017; Chapuy et al., 2018; Papageorgiou et al., 2021). C3 which was characterized by *BCL2* and chromatin modifier mutations, C4 was defined by mutations in four linker and four core histone genes, and these two pathways are mentioned together because they both represent subtypes of GCB type DLBCL (Chapuy et al., 2018). These subtypes are of interventional significance because they both alter the function of common pathways such as PI3K, but do so through different mechanisms (Chapuy et al., 2018). This is yet another example of why therapies must be tailored to the underlying mechanism of disease state, not just the presentation of disease. C2 was defined by markers including *TP53* mutation and loss of *CDKN2A* and *RB1* which alter chromosomal stability and cell cycle (Chapuy et al., 2018). Cluster zero (C0) lacked any defining characteristics or identifiable genetic homogeneity and represents a significant lack of understanding in disease pathology (Chapuy et al., 2018).

#### 2.2.3 Lacy’s classification: NOTCH2, BCL2, TET2/SGK1, SOCS1/SGK1, and MYD88

Research conducted by Lacy et al. (2020) sought to investigate their own set of subgroups using methods “potentially applicable in routine clinical medicine” and came up with 5 distinct groups. Their study aimed not only to establish groups with potentially workable mutations, but also to compare overlap between their groups to subtypes identified in studies Schmitz et al. and Chapuy et al. (2018) (Table 1). The first group, “MYD88” cluster, was defined by *MYD88* mutation among others and contained the majority of observed primary CNS lymphomas and primary testicular lymphomas, similarly to MCD and C5 groups (Schmitz et al., 2018; Lacy et al., 2020). The “BCL2” group predominantly had characteristic t(14;18) translocation and other mutations in BCL2 pathways (Lacy et al., 2020). “SOCS1/SGK1” had mutations common to primary mediastinal B-cell lymphoma, and was observed to represent a subdivision of the C4 cluster described by Chapuy et al. (2018) (Lacy et al., 2020). The “TET2/SGK1” was largely GCB in origin and had characteristic mutations including *TET2*, *SGK1*, and *KRAS*, and was postulated to represent another subdivision of the C4 cluster (Chapuy et al., 2018). The final subtype described here which corresponded to the C1 subtype was the “NOTCH2” subtype which included mutations in *NOTCH2*, *BCL10*, *CD70*, and showed a strong correlation between *NOTCH2* mutation and *BCL6* rearrangement (Chapuy et al., 2018; Schmitz et al., 2018; Lacy et al., 2020). These subtypes seem to provide some prognostic ability with 5-year OS rates for MYD88, SOCS1/SGK1, BCL2, TET2/SGK1, and NOTCH2 groups observed at 42.0%, 64.9%, 62.5%, 60.1%, and 48.1% respectively (Lacy et al., 2020). This study demonstrated significance not only for itself, but helped cement the significance of studies conducted by Schmitz et al. and Chapuy et al. stemming from the overlap of their respective subtypes in terms of genetic anomaly identified and observed disease course, especially in the context of different algorithms and research parameters utilized between the studies.

#### 2.2.4 Wright’s classification: BN2, A53, EZB-MYC+, EZB-MYC–, ST2, MCD, N1

Another key classification system using an algorithm referred to as “LymphGen” developed by Wright et al. (2020) intended on developing a more clinically useful stratification system based partially on work done by Schmitz et al. and Chapuy et al. This algorithm again recognizes genetic “constellations” rather than individual abnormalities and divides patients into 7 groups based on the prevalence of defined features. These groups are “MCD” characterized by BCR-dependent NF-κB immune evasion, “N1” characterized by NOTCH1 signaling and altered B cell differentiation, “A53” characterized by TP53 inactivation and aneuploidy, “BN2” characterized by NOTCH2 signaling with BCR dependent NF-κB immune evasion and a loss of CD70, “ST2” characterized by JAK/STAT3 signaling with NF-κB activation, and EZB which was further subdivided as being MYC+/− (“EZB-MYC+” and “EZB-MYC–“) and had chromatin modification conferring anti-apoptosis as well as PI3 kinase signaling and S1PR2-GNA13 inactivation (Wright et al., 2020). The LymphGen algorithm, using these criteria among other genetic markers, used genetic information gathered from malignant cells to then score the cells on the presence of these various mutations and assign them to the appropriate group based on assigned confidence intervals (Wright et al., 2020). The benefit to this algorithm was the ability to not only define subtype based on presence of these traits, but also to identify cells that had a probability of being part of a core, extended or genetically composite group where probability of belonging to a subtype was >90%, 50%–90%, or if the cell was a core member of more than one subtype, respectively (Wright et al., 2020). Using the algorithm, 63.1% of the cohort was able to be classified, greater than the 46.6% classified by Schmitz et al. (Wright et al., 2020). This is an especially high number when considering that to fall into one of the 7 subtypes with acceptable confidence for the algorithm, cells must acquire multiple mutations that seem as though they statistically should not occur together as often as they do if they were independent of each other. The ability to group these cells together may stem from a founder effect where a cell acquires an initial genetic mutation that may be relatively more common, and only certain secondary mutations confer continued survival for the cell. MYC overexpression would normally lead to cell death if not also paired with a *BCL2* translocation that prevents cell death (Evan et al., 1992). A sort of natural selection such as this confers the ability of these algorithms to become of clinical use in the near future since these mutations likely do not occur in isolation (e.g., *BCL2* and *MYC* mutations codependency), but are dependent on each other for the survival of the cell inextricably linking the altered pathways.

## 3 Genetic alterations in DLBCL

Understanding which genetic anomalies tend to occur together and the associated prognosis is an essential primary step in establishing a new categorization system for DLBCL, but is far from the end product. To develop effective treatments we must thoroughly understand the pathways that have undergone alteration, and establish whether or not they are even influential in overall disease progression. Equally important is establishing where in a specific pathway the disruption occurs, because mutations downstream of drug targets may render the drug useless, even if it acts on the appropriate pathway. By taking a closer look at which pathways tend to undergo alteration and where alterations tend to take place, we will establish not only the extreme complexity of DLBCL, but can begin to identify potential drug targets for later discussion of therapeutic indications.

### 3.1 Major signaling pathways affected by genetic alteration in DLBCL

**FIGURE 2:**
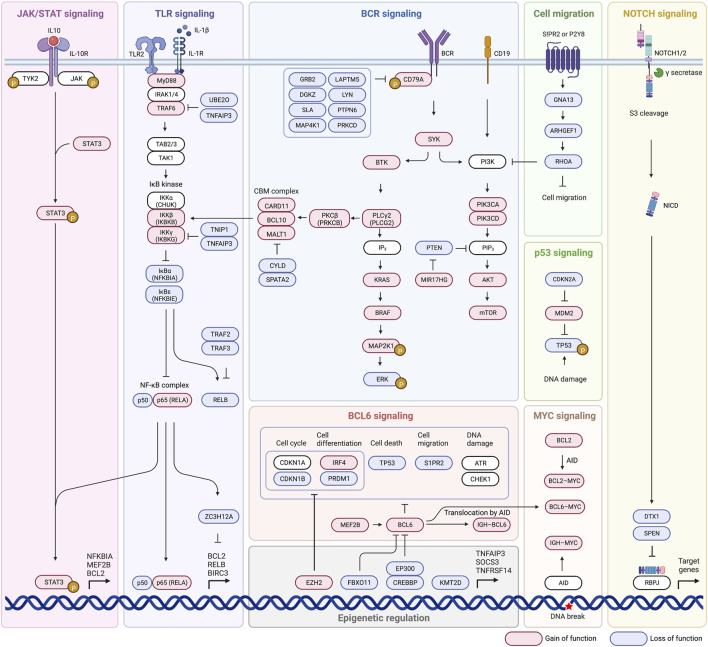
Major signaling pathways affected by genetic alterations in DLBCL.

Until now we have discussed pathways that have been identified as influential in disease state and developing criteria for new subtypes. We will now take a closer look at some of the most commonly mutated pathways, including BCR signaling, PI3K-AKT-mTOR signaling, BCR dependent NF-κB activation, NF-κB signaling, TLR signaling, and the BCL2 anti-apoptotic family (Figure 2). These pathways are related in their ability to evade apoptotic pathways, promote cell proliferation and gene expression, and confer lymphomagenesis. The ability to target not only faulty pathways, but specific faulty mutations within pathways, will pave the way for targeted DLBCL treatment, and identification of influential mutations is the first step in that direction.

#### 3.1.1 B-cell receptor (BCR) signaling

BCR signaling is involved in regulation of B cell survival, development, and differentiation, and can be chronic active or tonic in DLBCL, and both overexpression and overactivation are implicated in lymphomagenesis (Rawlings et al., 2017). Chronic signaling resembles antigen-dependent (autoreactive self-antigens) active BCR signaling in non-malignant B cells and is characterized by BCR clustering (Davis et al., 2010; Young and Staudt, 2013; Young et al., 2015; Havranek et al., 2017). Tonic BCR signaling is antigen-independent and clustering is not observed (Davis et al., 2010). ABC cells are characterized by chronic active BCR signaling, GCB DLBCL display tonic signaling (Davis et al., 2010; Havranek et al., 2017). During normal signaling after the BCR is bound by an appropriate ligand, CD79A and CD79B form a heterodimer and following dual phosphorylation of the intracellular immunotyrosine-based action motifs (ITAMs) of CD79A/CD79B, SYK kinase is recruited and activated which then activates BTK (Bruton’s tyrosine kinase) which leads to downstream activation of BCR signaling, notably mTOR and NF-κB (Miao et al., 2019). Mutations in any part of this pathway can potentially be problematic and lead to overactivation, such as loss of negative feedback ITAM mutation of *CD79B* which is detected in near 20% of ABC DLBCL cases (Davis et al., 2010). B cell receptors can be positively regulated by mutation or amplification of *CD79B* and *CD79A*, and negatively regulated by inhibitors including LAPTM5, LYN, PTPN6, GRB2, PRKCD, DGKZ, SLA, and MAP4K1 (Lenz et al., 2008a; Lam et al., 2008; Affymetrix® White Paper, 2009; Dobin et al., 2013; Anders et al., 2015; Seshan, 2017; NCI Genomic Data Commons, 2016). Loss of function of negative regulators of BCR signaling leads to unchecked activation, or a loss of negative feedback, of the BCR and was present in 38.5% of cases of DLBCL (Schmitz et al., 2018). Loss of function of negative regulators was more prevalent in *MYD88* with *CD79A*/*CD79B* activating mutations, than in *MYD88* lacking *CD79A*/*CD79B* alterations (56% vs 36.4%) which is of potential clinical utility because aggressive lymphomas with *MYD88* ^L265P^ and *CD79B* mutations have a response to ibrutinib (Bruton’s tyrosine kinase is downstream of BCR) and presumably have chronic active BCR signaling (Shaffer et al., 2006; Puente et al., 2015; Chapuy et al., 2016; Schmitz et al., 2018). The most common mutation affecting BCR and TLR signaling is *MYD88*, 18%–27% of cases, which directly affects TLR signaling and indirectly influences BCR signaling because MYD88, TLR9, and BCR form a multiprotein supercomplex which leads to promotion of downstream NF-κB and mTOR activation (Phelan et al., 2018).

#### 3.1.2 PI3K-AKT-mTOR signaling

PI3K can indirectly activate NF-κB pathway and was genetically altered in 34.3% of cases of DLBCL, and is also able to directly activate mTOR mechanisms (Kent, 2002). Activation of mTOR inappropriately promotes cell proliferation and metabolism that contributes to tumor initiation and progression (Tian et al., 2019). Normally, activation through the BCR or CD19 leads to activation of the PIK3CD/PIK3CA heterodimer (BCR does this indirectly through intermediate activation of PIK3AP1), which goes on to activate PIP3 leading to activation of PDPK1, then activation of an AKT1/AKT2 heterodimer, and final activation of mTOR (Schmitz et al., 2018; Miao et al., 2019). Activating events involving PI3K signaling subunits (PIK3CA, PIK3CD, PIK3AP1, PDK1, AKT1/2) can lead to downstream AKT activation (Miao et al., 2019). Activation of MIR17HG which functions to inhibit PTEN, a negative regulator of PIP3, also leads to overactivation of this pathway and overexpression of mTOR (Miao et al., 2019). Inactivating mutations of negative PIP3 regulators *PTEN* and *INPP5D* are also seen in up to 15% and 10% of cases respectively and lead to unchecked mTOR expression (Schmitz et al., 2018). BCR co-receptor CD19 and the BCR itself (through SYK kinase) are able to activate PI3K signaling, which leads to activation of AKT which activates downstream mTOR promoting cell survival, again showing the large role BCR signaling plays in promotion of DLBCL (Uddin et al., 2006; Young and Staudt, 2013). Activating mutations of PI3K subunits (*PIK3CA* amplification/activating mutation) are identified in 6% of ABC DLBCL but not GCB, others (*PTEN* deletion) are present in both ABC and GCB (9%–11%) (Lenz et al., 2008a; Pfeifer et al., 2013; Chapuy et al., 2018; Schmitz et al., 2018; Wang et al., 2018).

#### 3.1.3 BCR-dependent NF-κB activation

Normally, BCR stimulation caused by antigen binding activates BTK signaling through SYK activation, which activates PLCλ2 and PKCβ, PKCβ then phosphorylates CARD11 which recruits BCL10 and MALT1 to form a complex (CBM complex) that inactivates negative regulator CYLD/SPATA2 complex and inactivates RELB (Schmitz et al., 2018). ZC3H12A is a more independent regulator of NF-κB pathway-dependent transcripts and enhances mRNA decay of anti-apoptotic gene transcripts including *BCL2L1*, *BCL2A1*, *RELB*, *BIRC3*, and *BCL3* (Skalniak et al., 2009; Lu et al., 2016). Inactivating mutations of *ZC3H12A* are seen in up to 10% of cases, the consequence of which is anti-apoptotic and pro-lymphomagenesis (Skalniak et al., 2009; Lu et al., 2016; Schmitz et al., 2018). Failure of the NF-κB pathway to produce adequate IL-1β, which ZC3H12A is dependent on for activation, may also lead to inadequate mRNA degradation and anti-apoptotic characteristics (Skalniak et al., 2009). Mutations that cause overactivation of BCR signaling such as activating mutations of *CD79A*/*CD79B* or *SYK* which overstimulate BTK/PLCG2 causing activation of PRKCB and the CBM complex lead to an overactive NF-κB pathway (Schmitz et al., 2018). *CARD11* mutations, which occur in both ABC and GCB type, impair auto-inhibition seen in wild type proteins which lead to continued activation of NF-κB (Lenz et al., 2008b; Lamason et al., 2010; Wilson et al., 2012; Bohers et al., 2014; Schmitz et al., 2018). Inactivating mutations are also seen in the negative regulators of the CBM complex themselves, including inactivation of CYLD/SPATA2 complex which functions as the inhibitor of the CBM complex in a negative feedback loop (Schmitz et al., 2018). Inactivation of negative regulators of RELB is also seen in up to 15% of cases, specifically inactivation of the TRAF2/3 complex, and overactivation of RELB is thought to indicate dismal outcome after immunochemotherapy because RELB confers DLBCL cell resistance to DNA damage induced apoptosis, preventing genotoxic agent doxorubicin from having the desired effect (Schmitz et al., 2018; Eluard et al., 2022). Negative regulators of inactivation signaling enzymes and adaptors that promote BCR-dependent NF-kB activation are aberrant in 44.9% of cases (Schmitz et al., 2018). 66.2% of cases also had mutations in other regulators of NF-κB such as *TLR2* and *ZC3H12A* which negatively regulate the stability of NF-κB messenger RNA (mRNA) (Nicorici et al., 2014). In the BN2 subtype 2 components of BCR-dependent NF-κB pathway were altered in 47% of cases, protein kinase C beta (PKCB) and BCL10 (Schmitz et al., 2018). Signatures of BCR-dependent NF-κB activation were highest in MCD and BN2 (Schmitz et al., 2018).

#### 3.1.4 NF-κB signaling (IκB kinase-dependent NF-κB activation)

NF-κB signaling may also be stimulated through activation of the IKBKG/IKBKB complex, which is only partially dependent on the BCR pathway and may be activated through various means (Schmitz et al., 2018). Activating mutations of positive regulators, *TLR2* and *MYD88*, or inactivation of negative regulators TNIP1/TNFAIP3, result in the ability of IκB kinase (IKBKG/IKBKB) to inhibit the NFKBIA/NFKBIE complex (Schmitz et al., 2018; Miao et al., 2019; Ma and Malynn, 202012). Inhibition of this complex causes release of p65/p50 and REL pathways, which may also adopt activating mutations themselves, and final overexpression of NF-κB expression (Schmitz et al., 2018; Miao et al., 2019). Genetic alterations of NF-κB negative regulators were a prominent feature of subtype BN2, those affecting negative regulators TNFAIP3 or TNIP1 were found in 55% (Schmitz et al., 2018). Mutations in positive regulator *MYD88* are also a defining trait of the MCD subtype and are a prominent source of gene overexpression (Schmitz et al., 2018).

#### 3.1.5 Toll-like receptor (TLR) signaling

Toll-like receptor 2 (TLR2) signaling in its standard state is mediated by MYD88, an adapter protein which assists in signaling, in complex with IRAK1/4 (Ngo et al., 2011). Mutation of *MYD88* promotes assembly of a complex composed of IRAK1 and IRAK4 which enhances IRAK4 kinase activity and IRAK1 phosphorylation. Hyperphosphorylated IRAK1 causes activation of TRAF6, which activates TAK1, which can then accomplish downstream NF-κB and JAK/STAT signaling through the IκB kinase pathway (Ngo et al., 2011; Schmitz et al., 2018; Miao et al., 2019). Activating mutations of *TLR2*, *MYD88*, and *TRAF6* are observed and can stimulate this pathway in absence of a proper TLR ligand (Schmitz et al., 2018). Activating mutations of *MYD88* increase activity of both NF-κB and JAK/STAT pathways (Rosenquist et al., 2017). Inactivating mutations of negative regulators UBE2O and TNFAIP3, which normally inhibit TRAF6, are also observed to cause overstimulation of downstream NF-κB pathway (Miao et al., 2019).

#### 3.1.6 Anti-apoptotic BCL2 family

In healthy B cells, apoptosis is induced through a lack of affinity of BCL2 for a specific antigen, so constitutive BCL2 activation through mutation would result in B cells that can avoid apoptotic programs in the absence of proper antigen recognition (Miao et al., 2019). Various *BCL2* mutations occur and are noted in various DLBCL subtypes, such as the t(14;18)(q32;q21) translocation seen in 34%–44% of GCB DLBCL (Iqbal et al., 2004; Visco et al., 2013; Ennishi et al., 2017). Translocations such as this move *BCL2* near genes that cause overactivation, or constitutive activation of BCL2 and promote survival (Kridel et al., 2012). Somatic hypermutation involving promoter and coding regions of *BCL2* is seen in about 35% of DLBCL cases, with the majority being in the GCB subtype (Schuetz et al., 2012). These two examples indicate that while activation/mutation of *BCL2* is common across many DLBCL’s, the root cause is not the same and may drive the need for multiple approaches to treating the same faulty pathway. Different mutations also result in differential response to BCL2 inhibitors, which is a consideration in measuring response to BCL2 inhibitors (Schmitz et al., 2018). Overactivation of other BCL2 family members such as BCL2L1 and MCL1 can also impair intrinsic apoptotic pathways and lead to continued cell survival and proliferation (Schmitz et al., 2018; Miao et al., 2019).

### 3.2 Alterations in epigenetic regulation that contribute to the development of lymphoma

Previously discussed are mutations within genes themselves, but equally important are changes in epigenetic regulators that alter expression of these genes. Greater epigenetic heterogeneity is associated with poor clinical outcome (De et al., 2013; Jiang and Melnick, 2015) and inhibitors of these mechanisms such as DNA methyltransferase and histone methyltransferase inhibitors may be a source of therapeutic intervention in DLBCL (Jiang and Melnick, 2015). Each subtype may express epigenetic dysregulation in relatively characteristic ways, such as EZB type displaying *EZH2* mutations or inactivation of KMT2D (Schmitz et al., 2018). We will now take a closer look at disruptions in regulation caused by EZH2, KMT2D, and EP300 and CREBBP.

#### 3.2.1 EZH2

EZH2 is an important mediator of negative transcriptional activity, achieved through trimethylation of Lys27 of histone H3 subunit, referred to as H3K27 (Velichutina et al., 2010; Beguelin et al., 2013; Caganova et al., 2013). Overactivation of EZH2 leads to excessive activation of cell cycle regulators and promoters of plasma cell differentiation, such as CDKN1A, CDKN1B, CDKN2A, PRDM1, IRF4, or XBP1 (Miao et al., 2019). These problems culminate in defective germinal center formation in GCB cells, cells that can proliferate unchecked by the cell cycle regulators, protection from AID-dependent genotoxic damage-induced apoptosis, and restricted differentiation (Velichutina et al., 2010; Beguelin et al., 2013; Caganova et al., 2013). *EZH2* mutation alone is not enough to create an environment conducive to the development of DLBCL, however in mice it has been shown that *EZH2* mutation coupled with overexpression of BCL2 may be able to propagate the development of DLBCL (Cattoretti et al., 2005; Miao et al., 2019).

#### 3.2.2 KMT2D

KMT2D is a histone monomethyltransferase responsible for regulation of transcription of tumor suppressor genes such as *TNFAIP3*, *SOCS3*, or *TNFRSF14*, so inactivation of this gene will lead to decreased tumor suppressor activity (Ortega-Molina et al., 2015; Miao et al., 2019). Nearly 30% of DLBCLs show mutation in this gene, commonly a non-sense or frameshift, but some cases of DLBCL show decreased KMT2D activity in the absence of mutation indicating other epigenetic regulation may be relevant (Morin et al., 2011; Ortega-Molina et al., 2015; Schmitz et al., 2018). KMT2D inactivation is also implicated in disruption of cell cycle regulators CDK6 and BCL2, which promote malignant cell survival (Zhang et al., 2015).

#### 3.2.3 EP300 and CREBBP

Among multiple histone lysine acetylation sites, H3K27 acetylation is related to active transcription while deacetylation represses transcription. EP300 and CREBBP are involved in H3K27 acetylation to promote transcription of tumor suppressor genes such as *PRDM1*, *IRF4*, *CIITA*, *CD74*, or *HLADR*, as well as acetylation of BCL6 and p53 which results in inhibition and activation of transcription respectively (Miao et al., 2019). *CREBBP* mutation reduces expression of these genes essential to plasma cell differentiation and immune responses against lymphomagenesis, which can be found in about 20% of patients with DLBCL and more commonly in the GCB than ABC subtype (Pasqualucci et al., 2011a; Lohr et al., 2012). *CREBBP* mutations have been observed in mice to promote substantial increases in the number of germinal center B cells which promotes development of MYC-driven lymphoma (Hashwah et al., 2017). Deficiencies in CREBBP also reduce expression of genes responsible for germinal center exit, antigen presentation and immune response, promoting lymphomagenesis (Jiang et al., 2017). *EP300* is mutated in roughly 10% of DLBCLs of both ABC and GCB types, and effects are similar phenotypically to *CREBBP* mutations (Pasqualucci et al., 2011b).

### 3.3 Alterations in other pathways

Various other pathways are involved in the continued survival, proliferation, and immune evasion of malignant DLBCL cells. Subtype N1 is based off alterations in NOTCH signaling, germinal center homing pathways and migration are disrupted (S1PR2 and GNA13) in 38% of EZB type cases. BCL6 signaling disruption is also found in commonly in EZB subtype. *TP53* mutations prevent cell death, and *MYC* mutations are highly associated with MCD and BN2 type (Davis et al., 2010; Schmitz et al., 2018). Evasion of immune surveillance is seen across numerous DLBCL subtypes, and we will now take a closer look at these pathways.

#### 3.3.1 NOTCH signaling

NOTCH genes (*NOTCH1* and *NOTCH2*) code for a series of receptors involved in specialization of cells, cell growth, differentiation, and apoptosis (Notch1 Gene MedlinePlus, 2015). Mutations in *NOTCH1* are a defining characteristic of the N1 subtype, and *NOTCH2* mutations in combination with BCL6 fusions are defining of the BN2 subtype (Schmitz et al., 2018). Mutations in NOTCH1/2 signaling can be activating type in the NOTCH receptor itself or activating mutations of downstream intracellular messengers such as PEST, which can activate nuclear gene transcription (Lee et al., 2009; Arcaini et al., 2015). Inactivating mutations of *SPEN* and *DTX1*, negative regulators of NOTCH signaled transcription, could also lead to overexpression of genes (Schmitz et al., 2018; Miao et al., 2019).

#### 3.3.2 Cell migration

GCB cells under normal conditions should exist only within appropriate germinal centers but are found in circulation and extranodal sites in DLBCL (Montesinos-Rongen et al., 2011; Pham-Ledard et al., 2012; Pham-Ledard et al., 2014a; Pham-Ledard et al., 2014b; Kraan et al., 2014; Oishi et al., 2015; Chapuy et al., 2016; Fukumura et al., 2016; Taniguchi et al., 2016; Cao et al., 2017; Franco et al., 2017; Zheng et al., 2017; Zhou et al., 2018; Miao et al., 2019). Migration of these cells can likely be traced back to inactivation or under-activation of the sphingosine 1-phosphate receptor-2 (S1PR2) (Green et al., 2011; Muppidi et al., 2014; Miao et al., 2019). Under malignant conditions, S1PR2 is inactivated, resulting in a failure of activation of downstream GNA13, which then is unable to activate ARGHEF1, which then cannot activate RHOA, causing a lack of inhibition of cell migration (Muppidi et al., 2014; Rosenquist et al., 2017; Miao et al., 2019). *GNA13* and *ARHGEF1* normally encode for mediators that control the growth of GCB cells and confine them to germinal centers, so a lack of these mediator’s results in their systemic distribution and uncontrolled migration (Morin et al., 2011; Muppidi et al., 2014). The homing pathway encoded by *GNA13* and *ARHGEF1* is disrupted in 38% of EZB subtype cases, showing the importance of this specific pathway (Schmitz et al., 2018).

#### 3.3.3 BCL6 signaling

BCL6 fusions are commonly associated with *NOTCH2* mutations, which make up the defining characteristics of the BN2 subtype (Schmitz et al., 2018). BCL6 in its normal functional capacity is responsible for recruiting histone deacetylase 3 (HDAC3) which deacetylates BCL6-bound transcriptional enhancers, rendering them inaccessible for transcription (Hatzi et al., 2013; Miao et al., 2019). Alterations in the first-noncoding exon of BCL6 also interfere with negative autoregulation, further increasing activity of the gene (Miao et al., 2019). BCL6 regulates plasma cell differentiation (IRF4, PRDM1), cell migration (S1PR1), DNA damage response (ATR, CHEK1), the cell cycle (CDKN1A, CDKN1B), and cell death (TP53) (Miao et al., 2019). Activating mutations of *BCL6* (or activators such as *MEF2B*) or inactivating mutations of BCL6 inhibitors (*EP300*, *CREBBP*, *FBXO11*) lead to suppression of these processes previously mentioned, and promotion of germinal center formation and disruption of plasma cell formation (Cattoretti et al., 2005; Pasqualucci et al., 2011b; Miao et al., 2019).

#### 3.3.4 p53 signaling

p53, encoded for by *TP53*, plays a critical role in activation of genes involved in regulation of cell death and cell cycle progression, including *BAX* and *CDKN1A* (Miao et al., 2019). Inactivation of p53 through deletion of *TP53* is seen in 8%–24% of DLCBL (Jardin et al., 2010) but inactivation can occur through numerous other mechanisms. p14^ARF^ (CDKN2A) normally inhibits MDM2, preventing inactivation of p53 by MDM2, but p14^ARF^ can develop an inactivating mutation leading to constitutive inhibition of p53 by MDM2 (Schmitz et al., 2018; Miao et al., 2019; Lenz et al., 2008a; Ma and Malynn, 202012). Activating mutations of *MDM2* or *MDM4* (another inhibitor of p53) can also lead to continued inactivation of p53, and failure of *CDKN1A*/*BAX* mRNA transcription (Miao et al., 2019). Inactivation of p53 also causes failure of proper cell cycle progression (limited BTG1, BTG2, CDKN1A expression), failure of apoptotic pathways (limited FAS, BCL2 expression), and failure of adequate DNA repair mechanisms (limited BRCA2, ATM, PRKDC expression) (Rosenquist et al., 2017).

#### 3.3.5 MYC signaling

MYC is a proto-oncogene at the center of multiple second messenger pathways and regulates transcription of genes involved in cell growth and proliferation, apoptosis, metabolism, DNA replication, and protein biosynthesis (Dang, 2012; Karube and Campo, 2015). Cells with gene expression characteristic of MYC, and characteristic of proliferation, are highly associated with the MCD and BN2 subtypes (Schmitz et al., 2018). DLBCL with rearrangements involving *MYC* and *BCL2*/*BCL6*, which upregulate *MYC* gene expression, are referred to as double hit or triple hit lymphomas, and are associated with inferior progression free survival and overall survival in patients receiving traditional R-CHOP therapy (Hummel et al., 2006; Savage et al., 2009; Valera et al., 2013; Rosenquist et al., 2017). Interestingly however, *MYC* rearrangements are not predictive of poorer prognosis in patients who receive the DA-EPOCH-R regimen (etoposide, prednisone, vincristine, cyclophosphamide, doxorubicin, rituximab) suggesting the possible targetability of this pathway (Lai et al., 2018). Another study found MYC partner to be prognostically significant, with *IGH*-*MYC* translocations having the worst prognosis of all *MYC* translocations, likely because *IGH*-*MYC* translocations place *MYC* adjacent to immunoglobulin enhancers which leads to constitutive MYC expression (Copie-Bergman et al., 2015; Karube and Campo, 2015). One of the causes of *MYC* translocations with *IGH* or *BCL6* is thought to be mediated by activation-induced cytidine deaminase (AID), which normally is responsible for somatic hypermutation and class switch recombination, but can also target and create breaks in *BCL6* and *MYC* (Shen et al., 1998; Miao et al., 2019). Mutations resulting from incorrect AID-related hypermutation, especially in regions not coding for immunoglobulin genes, has been reported in more than half of DLBCL patients (Gordon et al., 2003; Deutsch et al., 2007; Miao et al., 2019). In addition to activating *MYC* translocations leading to downstream gene upregulation, inactivating mutations of the negative regulator MAX-gene associated protein (MGA) are also implicated in improper MYC expression (Miao et al., 2019). Not only are the MYC aberrations themselves relevant clinically as indicated by trials with DA-EPOCH-R protocol, but being able to further subtype these based on MYC partner or mutation location may provide further prognostic significance (Copie-Bergman et al., 2015; Karube and Campo, 2015; Xu-Monette et al., 2016).

#### 3.3.6 Evasion of immune surveillance

Immune editing and evasion of immune surveillance is a hallmark of the DLBCL, especially the MCD subtype and therefore the ABC type, with 76% of MCD acquiring mutation or deletion of *HLA-A*, *HLA-B*, or *HLA-C* (Chang et al., 2016). Inactivating alterations to MHC class I molecules, through augmentation or deletion of components HLA-A/B/C or β2 microglobulin (B2M) is common among DLBCL in both GCB and ABC types, and results in failed expression of surface MHC class I and subsequent failure to activate cytotoxic CD8^+^ T cells (Challa-Malladi et al., 2011). GCB types occasionally (10% of cases) also display disruptions in the MHC class II molecules, and thus failure to activate CD4^+^ T cells, through mutations in *HLA-DMA*/*HLA-DMB* or inactivating mutations in the MHC class II transactivator *CIITA* (Steimle et al., 1994; Schmitz et al., 2018). Natural killer (NK) cells display the CD2 receptor and are activated by the CD58 ligand, so mutations or deletions in *CD58* (21% of all DLBCL and in ABC 68% of the time) cause loss of this extracellular ligand and subsequent failure of NK-mediated cytolysis (Miao et al., 2019). CD4^+^ and CD8^+^ T cells also display the CD2 receptor, and activation would also be affected by loss of the CD58 ligand on the tumor cell (Miao et al., 2019). *CD274*/*PDCD1LG2* (PD-L1/PD-L2) gains, amplifications, and translocations are another mechanism by which DLBCL evades destruction by T cells, because binding of this ligand to PD-1 receptors on CD8^+^/CD4^+^ T cells prevents activation (Georgiou et al., 2016). Another deleterious mutation of DLBCL cells that can cause failure to activate immune cells is a loss of CD70, which would normally bind to CD27 on the CD4^+^ and/or CD8^+^ T cells to cause activation.

## 4 Targeted therapeutic strategies in DLBCL

**FIGURE 3:**
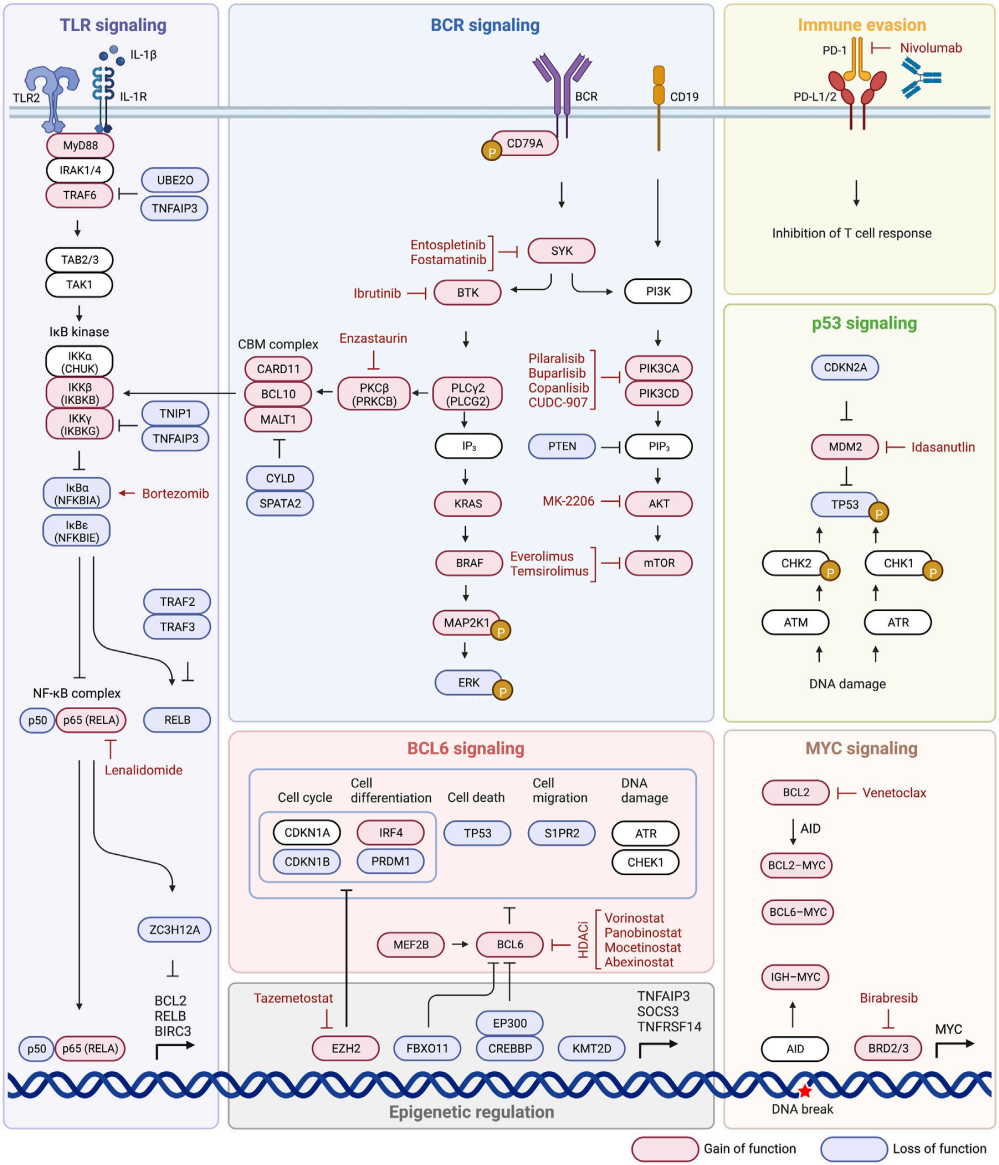
Targeted therapeutic strategies and potential inhibitors in DLBCL.

Having explored the various proposed ways to subtype DLBCL, some of the mechanisms behind the transformation of these malignant cells, and the various pathways that are associated with disease, it is now appropriate to delve in to targeted therapeutics. Acknowledging that DLBCL is a group of heterogenous disease processes that result in similar disease state and cellular phenotype, it is understandable that different therapies may be needed to target not only different pathways, but also the same pathway in different ways depending on how it was altered along its mechanism. While these various subtyping methods may not be entirely clinically useful or relevant yet, it is in our best interest to begin conceptually examining pharmaceuticals that could lead to outcomes superior to traditional R-CHOP therapy when applied to these experimental subgroups. We summarize drugs targeting BCR signaling, the PI3K-AKT-mTOR pathway, NF-κB signaling, BCL2 signaling, epigenetic pathways, MYC signaling, and finally immune evasion (Figure 3; Table 2).

**TABLE 2:** Potential therapeutic agents in DLBCL.

Pathway	Target	Therapeutic agent	Reference
NF-κB signaling	NFKBIA	Bortezomib	Chen et al. (2011); Goy et al. (2005); Offner et al. (2015); Leonard et al. (2017)
	RELA (p65)	Lenalidomide	Zhang et al. (2013); Czuczman et al. (2017)
BCR signaling	SYK	Entospletinib	Sharman et al. (2016)
	SYK	Fostamatinib	Friedberg et al. (2010)
	BTK	Ibrutinib	Wilson et al. (2012); Wilson et al. (2015); Younes et al. (2019)
	PRKCB	Enzastaurin	Robertson et al. (2007); Crump et al. (2016); Hainsworth et al. (2016)
PI3K-AKT-mTOR	PIK3CA/PIK3CD	Pilaralisib	Brown et al. (2015); Bechter et al. (2016)
	PIK3CA/PIK3CD	Buparlisib	Zang et al. (2014); Stewart et al. (2022)
	PIK3CA/PIK3CD	Copanlisib	Paul et al. (2017); Bojarczuk et al. (2019)
	PIK3CA/PIK3CD	CUDC-907	Oki et al. (2017)
	AKT	MK-2206	Oki et al. (2015)
	mTOR	Everolimus	Johnston et al. (2016); Witzig et al. (2017)
	mTOR	Temsirolimus	Major et al. (2022); Witzens-Harig et al. (2021)
Epigenetic pathway	EZH2	Tazemetostat	Radford et al. (2016); Italiano et al. (2018); Vincent Ribrag et al. (2018); Sarkozy et al. (2020)
	BCL6	Vorinostat (HDACi)	Siddiqi et al. (2020)
	BCL6	Panobinostat (HDACi)	Assouline et al. (2016)
	BCL6	Mocetinostat (HDACi)	Batlevi et al. (2017)
	BCL6	Abexinostat (HDACi)	Evens et al. (2016)
	BCL2	Venetoclax	Davids et al. (2017); de Vos et al. (2018); Morschhauser et al. (2021); Liu et al. (2018)
MYC signaling	BRD2/3	Birabresib	Delmore et al. (2011); Mottok and Gascoyne. (2015); Li et al. (2019); Amorim et al. (2016)
p53 signaling	MDM2	Idasanutlin	Gu et al. (2019)

### 4.1 Targeting BCR signaling

Three targets of the BCR signaling pathway that are actionable by current drugs, are PKCβ, SYK, and Bruton’s tyrosine kinase (BTK). Enzastaurin is a selective PKCβ inhibitor which would inhibit signal transduction and ultimate pathway activation, but efficacy has yet to be shown with this drug, and clinical failures have been attributed to mutations further down the pathway than at PKCβ (Robertson et al., 2007; Crump et al., 2016; Hainsworth et al., 2016). This drug may be of clinical use in patients with mutations specifically affecting PKCβ, so being able to detect mutations here would be essential in utilizing this treatment. SYK inhibitor (SYKi) entospletinib has shown promise in clinical trials following BTK or PI3Kδ inhibitors, with a response rate of 69% (Sharman et al., 2016). Fostamatinib is another SYKi but has shown little clinical benefit, and likely caused observed side effects (diarrhea, fatigue, nausea, hypertension, cytopenias) because it is a non-selective agent (Friedberg et al., 2010). Another promising agent in this pathway is ibrutinib, a Bruton’s tyrosine kinase inhibitor, and overall response rate in one monotherapy trial of refractory DLBCL with ibrutinib was 40% in ABC type and 5% in GBC type (Wilson et al., 2012; Intlekofer and Younes, 2014). This therapy is shown to disrupt the My-T-BCR complex with CARD11-BCL10-MALT1 complex and mTOR, with complete resistance in the ABC subtype provided by *CARD11* gene alterations (Lenz et al., 2008b; Phelan et al., 2018). MYD subtype in combination with *CD79A* and *CD79B* mutations increased susceptibility to ibrutinib, 80% of responses had *MYD88* mutation with concomitant *CD79B* mutation, while the wild type CD79A/CD79B provided protection from this intervention (Wilson et al., 2012; Wilson et al., 2015). One study showed addition of ibrutinib to traditional R-CHOP therapy increased event free survival and overall survival of patients under 60 years of age with non-GCB DLBCL, a notable finding as this is the first time addition of an agent to R-CHOP therapy has improved event free and overall survival of patients (Miao et al., 2019; Younes et al., 2019).

### 4.2 Targeting PI3K-AKT-mTOR

The PI3K-AKT-mTOR is another commonly active pathway in DLBCL with treatments that have shown promise in limited trials. CUDC-907 is a small molecule that inhibits PI3K (class Iα, β, and δ) and HDAC (class I and II), that showed a response rate of 64% in patients with DLBCL concurrent with MYC alteration (Oki et al., 2017). Other drugs targeting PI3K are pilaralisib (Brown et al., 2015; Bechter et al., 2016), buparlisib (Zang et al., 2014; Stewart et al., 2022), and copanlisib (Paul et al., 2017; Bojarczuk et al., 2019). AKT inhibitor MK-2206 showed promise in preclinical models, but failed to show results in any of the patients treated as part of a phase II trial (Oki et al., 2015). Two drugs showing some clinical significance are the mTOR inhibitors everolimus and temsirolimus, with positive responses observed, and one patient achieved a durable and complete response to the everolimus for years (Iyer et al., 2012; Milowsky et al., 2013; Witzens-Harig et al., 2021; Major et al., 2022). The patient was noted as the only participant in the trial with mutations in both *TSC1* and *NF2*, which were deemed significant to the dramatic efficacy of this drug (Iyer et al., 2012; Milowsky et al., 2013). Another cohort consisting of 24 patients who had not received any prior treatment for their DLBCL were given everolimus added to R-CHOP-21 induction therapy (R-CHOP given over 21 days) and demonstrated no disease progression or relapse after 24 months in all 24 patients (Johnston et al., 2016; Witzig et al., 2017). Further trials with the R-CHOP-21 plus everolimus will be needed to establish this as a superior modality to traditional R-CHOP therapy, but preliminary responses show promise (Johnston et al., 2016; Witzig et al., 2017).

### 4.3 Targeting NF-κB signaling

Lenalidomide acts on the NF-κB pathway by targeting the E3 ubiquitin ligase component of cereblon, and shows substantial activity in patients with relapsed or refractory DLBCL alone or in combination with other regimens (Wiernik et al., 2008; Hernandez-Ilizaliturri et al., 2011; Witzig et al., 2011; Zinzani et al., 2011; Hitz et al., 2013; Wang et al., 2013; Zhang et al., 2013; Feldman et al., 2014; Hitz et al., 2016; Martin et al., 2016; Czuczman et al., 2017; Ferreri et al., 2017). The greatest effects have been seen in patients with non-GCB or ABC DLBCLs (Wiernik et al., 2008; Hernandez-Ilizaliturri et al., 2011; Witzig et al., 2011; Zinzani et al., 2011; Hitz et al., 2013; Wang et al., 2013; Feldman et al., 2014; Hitz et al., 2016; Martin et al., 2016; Czuczman et al., 2017; Ferreri et al., 2017), and the addition of lenalidomide to CHOP treatment in patients with novel DLBCL seems to negate the negative prognostic implications of non-GCB DLBCL (Nowakowski et al., 2015). Lenalidomide has also been shown to be effective as a maintenance therapy and prolong PFS in patients aged 50–80 who respond to R-CHOP (Reddy et al., 2017; Thieblemont et al., 2017). Another mechanism for the utility of lenalidomide may be realized, aside from acting as a sole inhibitor of the NF-κB pathway, is through synthetic lethality (Kaelin, 2005). Synthetic lethality can be explained simply by the concept of redundancy, targeting of one gene or pathway alone has little to no effect on the cell while targeting both genes or pathways will result in cell death (Kaelin, 2005). This is seen in MYD88 DLBCLs where *MYD88* mutations promote NF-κB signaling, encouraging cell proliferation and survival, while also promoting the production of interferon-beta (IFNβ) which is cytotoxic to DLBCL cells (Yang et al., 2012). IFNβ is negatively regulated by interferon regulatory factor 4 and Spi-B in an inhibitory circuit that is reinforced by chronic BCR stimulation, so the cytotoxic effects of IFNβ are not seen (Yang et al., 2012). Lenalidomide can induce degradation of the inhibitory factors Interferon regulatory factor 4 (IRF4) and IFNβ, and ibrutinib is able to inhibit BCR signaling, resulting in increased IFNβ (through decreased degradation) and a synthetic lethality effect (Yang et al., 2012). This approach to synthetic lethality could prove to be clinically significant in trials, but identification of these synergistic pathways is trailing behind and must be further explored with approaches such as RNA interference screening (Kaelin, 2005). Another drug acting on the NF-κB path is bortezomib which downregulates NF-κB through inhibition of proteasomal degradation of IκBα, but is has failed to show significant efficacy as a monotherapy or in addition to R-CHOP therapy (Goy et al., 2005; Chen et al., 2011; Offner et al., 2015; Leonard et al., 2017).

### 4.4 Targeting BCL2 signaling

BCL2 expression is one of the most influential alterations in DLBCL and is already prognostically significant, as DLBCL with *MYC* and *BCL2*/*BCL6* mutations are termed double hit or triple hit lymphomas and are associated with a worse prognosis (Xu et al., 2013). Venetoclax is a BCL2 inhibitor that was shown to have a complete response in 12% of patients when used as a monotherapy, and an overall response rate of 41% when used in combination with bendamustine plus rituximab (Davids et al., 2017; de Vos et al., 2018). In patients with confirmed *BCL2* mutations venetoclax had a superior overall response rate when compared to R-CHOP in the matched population, suggesting the potential of venetoclax to improve outcomes in patients receiving R-CHOP (Morschhauser et al., 2021). One of the possible genes responsible for variable BCL2 inhibitor response is *PMAIP1*, *in vitro* studies have shown that DLBCL cells with amplifications of this gene are more sensitive to drugs like venetoclax (Liu et al., 2018). This further backs the notion that understanding the exact alteration in specific pathways plays a key role in treatment selection and response.

Another mechanism of therapeutic interest may be the inhibition of AID which functions as the driver of somatic hypermutation in B cells (Muramatsu et al., 2000). AID terminally leads to conversion of cytosine residues into uracil residues in single-stranded DNA, and is able to target not only immunoglobulin variable genes or switch recombination sequences, but also transcriptionally active genes such as *BCL6* and *MYC* (Robbiani and Nussenzweig, 2013). AID-mediated off-target mutations and subsequent double-stranded breaks are thought to be associated with oncogenic transformation (Lieber, 2016). Particularly, AID-dependent class-switch recombination is associated with *IGH*-*MYC* and *IGH*-*BCL6* translocations because breaks involve the switch region of *IGH* which is characteristic of AID (Lenz et al., 2007). Prevalence of mutations in *BCL2*, *SGK1*, *PIM1*, and *IGLL5* associated with AID were noted in a 2018 study, and predominance of single nucleotide substitutions help to confirm this association between mutations and overactive AID (Chapuy et al., 2018). Possible future therapies may aim to downregulate or completely inhibit the off-target mutations mediated by AID, and reduce the genetic burden on cells and prevent new, treatment-resistant prodigy cells from appearing and propagating.

### 4.5 Targeting epigenetic pathways

Gene regulation is another great target for manipulation since DLBCL displays frequent disruptions in histone-modifying enzymes and the general activity of genes (Jiang et al., 2017). Tazemetostat is an EZH2 inhibitor with an encouraging safety profile and response in both EZH2 wild-type and mutant relapsed/refractory (R/R) DLBCL, with responses up to 60% in R/R DLBCL (Radford et al., 2016; Italiano et al., 2018; Vincent Ribrag et al., 2018; Sarkozy et al., 2020). This drug was shown in one trial to be implicated in complete remission among all patients who received 8 complete cycles of tazemetostat in combination with traditional R-CHOP therapy (Sarkozy et al., 2020). Histone deacetylase inhibitors (HDACi) such as vorinostat (Siddiqi et al., 2020), panobinostat (Assouline et al., 2016), mocetinostat (Batlevi et al., 2017), and abexinostat (Evens et al., 2016) show potential benefit in certain patients with B-cell lymphoma when combined with other chemotherapies. Use of HDACi is proving useful especially in CREBBP-mutant cells, to restore acetylation of histones at transcriptional enhancer regions to enhance expression of tumor suppressor genes (Jiang et al., 2017). HDACi’s are also of interest in individuals with elevated MYC concurrent with elevated BCL2 levels, and can lead to induction of apoptosis through acetylated BCL6 accumulation (Bereshchenko et al., 2002; Duan et al., 2005; Kurland and Tansey, 2008; Zhang et al., 2012). Patients treated with panobinostat who had R/R DLBCL showed response rates as high as 67% when *MEF2B* mutations were also present, as opposed to 18% in those with wild type, indicating the potential benefit of these therapies (Assouline et al., 2016). Further studies including patients with p53 dysregulation may also be warranted, as these HDACi’s also increase levels of acetylated (wild-type) p53 which stimulates pro-apoptotic pathways (Dai and Gu, 2010). Additionally, MDM2 antagonist, idasanutlin, showed potent anti-tumor activity in both ABC and GCB cell lines and idasanutlin could be used as a novel drug in the clinical setting of DLBCL (Gu et al., 2019).

### 4.6 Targeting MYC signaling

The ability to block overactive *MYC* transcription is of interest because of the genes’ involvement in not only overall pathogenesis, but also its association with relapse and refractory DLBCL (Mondello et al., 2017). The bromodomain and extra-terminal (BET) protein family (BRD2, BRD3, BRD4, and BRDT) enhance *MYC* transcription by binding acetylated histones. BET inhibitors (BETi) interfere with BET-mediated *MYC* transcription through disruption of bromodomain-containing proteins which normally organize transcriptional machinery (Delmore et al., 2011; Mottok and Gascoyne, 2015; Li et al., 2019). Birabresib specifically is a drug of interest, and functions through binding to the BRD2 and BRD3, limiting the transcription of *MYC* among other oncogenes (Amorim et al., 2016). The suppression of these other oncogenes may require further exploration to understand the true mechanism of this drug, a phase II clinical trial with birabresib showed a 18% response and 12% complete response among eligible participants, without any apparent correlation with MYC expression (Amorim et al., 2016).

### 4.7 Targeting immune evasion

Evasion of DLBCL immune surveillance is accomplished through various mechanisms, but PD-L1 (*CD274*) and PD-L2 (*PDCD1LG2*) dysregulation is one of the most notable from a therapeutic standpoint. This pathway is targetable by antibodies that bind and block the PD-L1/2 ligand on the tumor cell, preventing it from binding PD-1/2 receptor and disabling immune escape (Miao et al., 2019). One such anti-PD-L1 antibody that can be administered is nivolumab, which has shown some promise in studies with ORR of 36% in patients with R/R DLBCL (Lesokhin et al., 2016). Failure to respond has been attributed to the relatively small number of patients in the study (16% and 3% respectively) who had 9p21.1 (*CD274*/*PDCD1LG2*) low level copy gains and amplifications (Ansell et al., 2019). An alternate study (KEYNOTE-013) that measured response in patients with and without *CD274* mutations showed a 50% response in patients with copy number gains, amplifications, and translocations of *CD274*, as opposed to a 9% response in those who did not have these mutations (Justin Kline et al., 2018). These results suggest what was proposed, that *CD274* mutations may be predictive of response to anti-PD-L1 antibodies (Justin Kline et al., 2018). A third study consisting of 4 patients with R/R primary CNS DLBCL and 1 patient with primary testicular DLBCL showed a response to nivolumab in 5/5 patients, and 3 patients that remained in continuous remission (Nayak et al., 2017). The success of this trial was attributed to *CD274*/*PDCD1LG2* gains, amplifications, and translocations that are commonly seen in patients with primary CNS DLBCL (60%) and primary testicular DLBCL (60%) and further emphasizes the importance of these PD-1 ligands in predicting PD-1 antibody treatment success (Chapuy et al., 2016; Nayak et al., 2017).

## 5 Conclusion and future perspectives

DLBCL encompasses a wide array of disease mechanisms and presentations that have historically been and are currently, lumped into essentially two prognostic groups all with the same therapeutic indication. Various novel ways to delineate cases of DLBCL have been proposed by groups such as Schmitz et al., 2018, Chapuy et al., 2016, Lacy et al., 2020, and Wright et al., 2020. These systems have used various algorithms to group DLBCL by “constellations” of genetic alteration instead of examining and grouping each alteration individually, because it is the sum of the parts that leads to the specific disease state and that may point to specific treatment regimen. These groups each had varying amounts of overlap in their classification systems, which may indicate the strength of the proposed subtypes as far as prevalence in the DLBCL population and impact of the established genetic constellation. Mutations in pathways regulating BCR signaling, the PI3K-AKT-mTOR signaling pathway, BCR-dependent NF-κB signaling, NF-κB signaling, TLR signaling, and the BCL2 family are among the most influential when it came to subdividing DLBCL cases into new subgroups. The end result of these pathways, whether it be overstimulation of pro-growth factors or inhibition of apoptotic pathways, leads to the same phenotypic result of continued cell growth and survival, yet cannot be treated as a singular problem. Even therapies directed specifically at these pathways may continue to fail if they treat steps further up the cascade than the mutation actually lies, so having the ability to identify and target multiple steps in these pathways will be a key factor in extending overall survival for these patients. Dysregulation of genes involved in epigenetic regulation such as *EZH2*, *KMT2D*, *EP300*, and *CREBBP* may also result in aberrant pathway activation or inactivation in many DLBCL cases, so exploring treatments directed at regulation of these pathways may also prove to be impactful on outcomes. Other pathways of interest with potential therapeutic interventions are the NOTCH signaling pathway, malignant cell migration resulting from S1PR2 inactivation, BCL6 signaling, p53 signaling, MYC signaling, and immune evasion through mutations in various receptors and ligands such as PD-L1 overexpression. Therapies currently showing promise include ibrutinib in targeting BCR signaling, everolimus in the PI3K-AKT-mTOR pathway, lenalidomide in targeting NF-κB signaling, venetoclax in BCL2 signaling, tazemetostat in EZH2 epigenetic regulation, birabresib in targeting MYC signaling, and nivolumab in targeting immune evasion.

In order to progress in the treatment of DLBCL, a new classification system must first be implemented as part of NCCN guidelines that will allow physicians to provide better prognostic information, and which may also indicate which second line therapies may have superior response when first line R-CHOP fails. Availability of NGS for use in patients with DLBCL will also need to be increased, as these groupings rely on this in combination with novel algorithms to appropriately place a malignancy in its respective group. Implementation of these groups will also greatly supplement data concerning tracking of disease course by group and response to therapies, because currently the population of DLBCL that has had their disease subtyped or genomically sequenced is relatively low and limited to experimental groups. With larger quantities of data we will be able to further differentiate which mutations are impactful on disease course, and which mutations may indicate a specific or targeted treatment. This will also allow us to increase the specificity of the subgroups and establish a more concrete stratification system. There is little doubt that a newly implemented subtyping system for DLBCL will need revision over time as data is collected, and the standard of care R-CHOP therapy will not be replaced overnight. Currently implementation of these groups will provide us with much needed and much lacking data and will give providers and patients the ability to supplement second- or third-line therapies with regimens targeted to their specific subtype or mutation which has never previously been done. Providing one more therapeutic option before making the move to palliative care is enough justification alone to support the use of new groups and targeted therapies and is step-one on the road to creating a new standard of care for these patients.

## References

[B30] Affymetrix® White Paper (2009). Copy number algorithm with built-in GC waviness correction in genotyping console™ software. Available at: https://www.affymetrix.com/support/technical/whitepapers/genotyping_console_copynumber_whitepaper.pdf (Accessed October 22, 2022).

[B1] Alaggio R. Amador C. Anagnostopoulos I. Attygalle A. D. Araujo I. B. O. Berti E. (2022). The 5th edition of the World Health organization classification of haematolymphoid Tumours: Lymphoid Neoplasms. Leukemia 36 (7), 1720–1748. Epub 2022/06/23. 10.1038/s41375-022-01620-2 35732829 PMC9214472

[B2] Amorim S. Stathis A. Gleeson M. Iyengar S. Magarotto V. Leleu X. (2016). Bromodomain inhibitor Otx015 in patients with lymphoma or multiple myeloma: A dose-escalation, open-label, pharmacokinetic, phase 1 study. Lancet Haematol. 3 (4), e196–e204. Epub 20160318. 10.1016/S2352-3026(16)00021-1 27063978

[B3] Anders S. Pyl P. T. Huber W. (2015). Htseq--a Python framework to work with high-throughput sequencing data. Bioinformatics 31 (2), 166–169. Epub 20140925. 10.1093/bioinformatics/btu638 25260700 PMC4287950

[B4] Ansell S. M. Minnema M. C. Johnson P. Timmerman J. M. Armand P. Shipp M. A. (2019). Nivolumab for relapsed/refractory diffuse large B-cell lymphoma in patients ineligible for or having failed autologous transplantation: A single-arm, phase ii study. J. Clin. Oncol. 37 (6), 481–489. Epub 20190108. 10.1200/JCO.18.00766 30620669 PMC6528729

[B5] Arcaini L. Rossi D. Lucioni M. Nicola M. Bruscaggin A. Fiaccadori V. (2015). The notch pathway is recurrently mutated in diffuse large B-cell lymphoma associated with hepatitis C virus infection. Haematologica 100 (2), 246–252. Epub 20141107. 10.3324/haematol.2014.116855 25381127 PMC4803124

[B6] Assouline S. E. Nielsen T. H. Yu S. Alcaide M. Chong L. MacDonald D. (2016). Phase 2 study of panobinostat with or without rituximab in relapsed diffuse large B-cell lymphoma. Blood 128 (2), 185–194. Epub 20160510. 10.1182/blood-2016-02-699520 27166360 PMC4972610

[B7] Batlevi C. L. Crump M. Andreadis C. Rizzieri D. Assouline S. E. Fox S. (2017). A phase 2 study of mocetinostat, a histone deacetylase inhibitor, in relapsed or refractory lymphoma. Br. J. Haematol. 178 (3), 434–441. Epub 2017/04/26. 10.1111/bjh.14698 28440559 PMC5576135

[B8] Bechter O. E. Dumez H. Costermans J. Punie K. Hsu K. Dedieu J. F. (2016). Phase I safety and pharmacokinetic dose-escalation study of pilaralisib polymorph E, a phosphoinositide 3-kinase inhibitor in tablet formulation, in patients with solid tumors or lymphoma. Cancer Chemother. Pharmacol. 78 (1), 83–90. Epub 2016/05/14. 10.1007/s00280-016-3056-0 27169794

[B9] Beguelin W. Popovic R. Teater M. Jiang Y. Bunting K. L. Rosen M. (2013). Ezh2 is required for germinal center formation and somatic Ezh2 mutations promote lymphoid transformation. Cancer Cell. 23 (5), 677–692. 10.1016/j.ccr.2013.04.011 23680150 PMC3681809

[B10] Bereshchenko O. R. Gu W. Dalla-Favera R. (2002). Acetylation inactivates the transcriptional repressor Bcl6. Nat. Genet. 32 (4), 606–613. Epub 20021028. 10.1038/ng1018 12402037

[B11] Bohers E. Mareschal S. Bouzelfen A. Marchand V. Ruminy P. Maingonnat C. (2014). Targetable activating mutations are very frequent in gcb and abc diffuse large B-cell lymphoma. Genes Chromosom. Cancer 53 (2), 144–153. Epub 20131105. 10.1002/gcc.22126 24327543

[B12] Bojarczuk K. Wienand K. Ryan J. A. Chen L. Villalobos-Ortiz M. Mandato E. (2019). Targeted inhibition of PI3Kα/δ is synergistic with BCL-2 blockade in genetically defined subtypes of DLBCL. Blood 133 (1), 70–80. Epub 2018/10/17. 10.1182/blood-2018-08-872465 30322870 PMC6318426

[B13] Brown J. R. Davids M. S. Rodon J. Abrisqueta P. Kasar S. N. Lager J. (2015). Phase I trial of the pan-pi3k inhibitor pilaralisib (Sar245408/Xl147) in patients with chronic lymphocytic leukemia (cll) or relapsed/refractory lymphoma. Clin. Cancer Res. 21 (14), 3160–3169. Epub 2015/04/05. 10.1158/1078-0432.CCR-14-3262 25840972

[B14] Caganova M. Carrisi C. Varano G. Mainoldi F. Zanardi F. Germain P. L. (2013). Germinal center dysregulation by histone methyltransferase Ezh2 promotes lymphomagenesis. J. Clin. Investig. 123 (12), 5009–5022. Epub 20131108. 10.1172/JCI70626 24200695 PMC3859423

[B15] Campo E. Jaffe E. S. Cook J. R. Quintanilla-Martinez L. Swerdlow S. H. Anderson K. C. (2022). The international Consensus classification of mature lymphoid Neoplasms: A report from the clinical advisory committee. Blood 140 (11), 1229–1253. Epub 2022/06/03. 10.1182/blood.2022015851 35653592 PMC9479027

[B16] Campo E. Swerdlow S. H. Harris N. L. Pileri S. Stein H. Jaffe E. S. (2011). The 2008 who classification of lymphoid Neoplasms and beyond: Evolving concepts and practical applications. Blood 117 (19), 5019–5032. Epub 20110207. 10.1182/blood-2011-01-293050 21300984 PMC3109529

[B17] Cao X. X. Li J. Cai H. Zhang W. Duan M. H. Zhou D. B. (2017). Patients with primary breast and primary female genital tract diffuse large B cell lymphoma have a high frequency of Myd88 and Cd79b mutations. Ann. Hematol. 96 (11), 1867–1871. Epub 20170812. 10.1007/s00277-017-3094-7 28803429

[B18] Cao Y. Zhu T. Zhang P. Xiao M. Yi S. Yang Y. (2016). Mutations or copy number losses of Cd58 and Tp53 genes in diffuse large B cell lymphoma are independent unfavorable prognostic factors. Oncotarget 7 (50), 83294–83307. 10.18632/oncotarget.13065 27825110 PMC5347770

[B19] Cattoretti G. Pasqualucci L. Ballon G. Tam W. Nandula S. V. Shen Q. (2005). Deregulated Bcl6 expression recapitulates the pathogenesis of human diffuse large B cell lymphomas in mice. Cancer Cell. 7 (5), 445–455. 10.1016/j.ccr.2005.03.037 15894265

[B20] Cerami E. Gao J. Dogrusoz U. Gross B. E. Sumer S. O. Aksoy B. A. (2012). The cbio cancer genomics portal: An open platform for exploring multidimensional cancer genomics data. Cancer Discov. 2 (5), 401–404. 10.1158/2159-8290.CD-12-0095 22588877 PMC3956037

[B21] Challa-Malladi M. Lieu Y. K. Califano O. Holmes A. B. Bhagat G. Murty V. V. (2011). Combined genetic inactivation of β2-Microglobulin and CD58 reveals frequent escape from immune recognition in diffuse large B cell lymphoma. Cancer Cell. 20 (6), 728–740. Epub 20111201. 10.1016/j.ccr.2011.11.006 22137796 PMC3660995

[B22] Chang M. T. Asthana S. Gao S. P. Lee B. H. Chapman J. S. Kandoth C. (2016). Identifying recurrent mutations in cancer reveals widespread lineage diversity and mutational specificity. Nat. Biotechnol. 34 (2), 155–163. Epub 20151130. 10.1038/nbt.3391 26619011 PMC4744099

[B23] Chapuy B. McKeown M. R. Lin C. Y. Monti S. Roemer M. G. Qi J. (2013). Discovery and characterization of super-enhancer-associated dependencies in diffuse large B cell lymphoma. Cancer Cell. 24 (6), 777–790. 10.1016/j.ccr.2013.11.003 24332044 PMC4018722

[B24] Chapuy B. Roemer M. G. Stewart C. Tan Y. Abo R. P. Zhang L. (2016). Targetable genetic features of primary testicular and primary central nervous system lymphomas. Blood 127 (7), 869–881. Epub 20151223. 10.1182/blood-2015-10-673236 26702065 PMC4760091

[B25] Chapuy B. Stewart C. Dunford A. J. Kim J. Kamburov A. Redd R. A. (2018). Molecular subtypes of diffuse large B cell lymphoma are associated with distinct pathogenic mechanisms and outcomes. Nat. Med. 24 (5), 679–690. Epub 20180430. 10.1038/s41591-018-0016-8 29713087 PMC6613387

[B26] Chen D. Frezza M. Schmitt S. Kanwar J. Dou Q. P. (2011). Bortezomib as the first proteasome inhibitor anticancer drug: Current status and future perspectives. Curr. Cancer Drug Targets 11 (3), 239–253. 10.2174/156800911794519752 21247388 PMC3306611

[B27] Coiffier B. (2007). Rituximab therapy in malignant lymphoma. Oncogene 26 (25), 3603–3613. 10.1038/sj.onc.1210376 17530014

[B28] Coiffier B. Sarkozy C. (2016). Diffuse large B-cell lymphoma: R-Chop failure-what to do? Hematol. Am. Soc. Hematol. Educ. Program 2016 (1), 366–378. 10.1182/asheducation-2016.1.366 PMC6142522 27913503

[B29] Copie-Bergman C. Cuilliere-Dartigues P. Baia M. Briere J. Delarue R. Canioni D. (2015). Myc-ig rearrangements are negative predictors of survival in dlbcl patients treated with immunochemotherapy: A gela/lysa study. Blood 126 (22), 2466–2474. Epub 20150915. 10.1182/blood-2015-05-647602 26373676

[B31] Crump M. Leppa S. Fayad L. Lee J. J. Di Rocco A. Ogura M. (2016). Randomized, double-blind, phase iii trial of enzastaurin versus placebo in patients achieving remission after first-line therapy for high-risk diffuse large B-cell lymphoma. J. Clin. Oncol. 34 (21), 2484–2492. Epub 20160523. 10.1200/JCO.2015.65.7171 27217449

[B32] Czuczman M. S. Trneny M. Davies A. Rule S. Linton K. M. Wagner-Johnston N. (2017). A phase 2/3 multicenter, randomized, open-label study to compare the efficacy and safety of lenalidomide versus investigator's choice in patients with relapsed or refractory diffuse large B-cell lymphoma. Clin. Cancer Res. 23 (15), 4127–4137. Epub 20170405. 10.1158/1078-0432.CCR-16-2818 28381416 PMC8171498

[B33] Dai C. Gu W. (2010). P53 post-translational modification: Deregulated in tumorigenesis. Trends Mol. Med. 16 (11), 528–536. 10.1016/j.molmed.2010.09.002 20932800 PMC2978905

[B34] Dang C. V. (2012). Myc on the path to cancer. Cell. 149 (1), 22–35. 10.1016/j.cell.2012.03.003 22464321 PMC3345192

[B36] Davids M. S. Roberts A. W. Seymour J. F. Pagel J. M. Kahl B. S. Wierda W. G. (2017). Phase I first-in-human study of venetoclax in patients with relapsed or refractory non-hodgkin lymphoma. J. Clin. Oncol. 35 (8), 826–833. Epub 20170117. 10.1200/JCO.2016.70.4320 28095146 PMC5455685

[B37] Davis R. E. Ngo V. N. Lenz G. Tolar P. Young R. M. Romesser P. B. (2010). Chronic active B-Cell-Receptor signalling in diffuse large B-cell lymphoma. Nature 463 (7277), 88–92. 10.1038/nature08638 20054396 PMC2845535

[B38] De S. Shaknovich R. Riester M. Elemento O. Geng H. Kormaksson M. (2013). Aberration in DNA methylation in B-cell lymphomas has a complex origin and increases with disease severity. PLoS Genet. 9 (1), e1003137. Epub 20130110. 10.1371/journal.pgen.1003137 23326238 PMC3542081

[B39] de Vos S. Swinnen L. J. Wang D. Reid E. Fowler N. Cordero J. (2018). Venetoclax, bendamustine, and rituximab in patients with relapsed or refractory nhl: A phase ib dose-finding study. Ann. Oncol. 29 (9), 1932–1938. 10.1093/annonc/mdy256 30060083 PMC6158762

[B40] Delmore J. E. Issa G. C. Lemieux M. E. Rahl P. B. Shi J. Jacobs H. M. (2011). Bet bromodomain inhibition as a therapeutic strategy to target C-myc. Cell. 146 (6), 904–917. Epub 20110901. 10.1016/j.cell.2011.08.017 21889194 PMC3187920

[B41] Deutsch A. J. Aigelsreiter A. Staber P. B. Beham A. Linkesch W. Guelly C. (2007). Malt lymphoma and extranodal diffuse large B-cell lymphoma are targeted by aberrant somatic hypermutation. Blood 109 (8), 3500–3504. Epub 20061229. 10.1182/blood-2006-06-030494 17197434

[B42] Dobin A. Davis C. A. Schlesinger F. Drenkow J. Zaleski C. Jha S. (2013). Star: Ultrafast universal rna-seq aligner. Bioinformatics 29 (1), 15–21. Epub 20121025. 10.1093/bioinformatics/bts635 23104886 PMC3530905

[B43] Duan H. Heckman C. A. Boxer L. M. (2005). Histone deacetylase inhibitors down-regulate bcl-2 expression and induce apoptosis in T(14;18) lymphomas. Mol. Cell. Biol. 25 (5), 1608–1619. 10.1128/MCB.25.5.1608-1619.2005 15713621 PMC549348

[B44] Eluard B. Nuan-Aliman S. Faumont N. Collares D. Bordereaux D. Montagne A. (2022). The alternative RelB NF-κB subunit is a novel critical player in diffuse large B-cell lymphoma. Blood 139 (3), 384–398. 10.1182/blood.2020010039 34232979

[B45] Ennishi D. Mottok A. Ben-Neriah S. Shulha H. P. Farinha P. Chan F. C. (2017). Genetic profiling of myc and Bcl2 in diffuse large B-cell lymphoma determines cell-of-origin-specific clinical impact. Blood 129 (20), 2760–2770. Epub 20170328. 10.1182/blood-2016-11-747022 28351934

[B46] Evan G. I. Wyllie A. H. Gilbert C. S. Littlewood T. D. Land H. Brooks M. (1992). Induction of apoptosis in fibroblasts by C-myc protein. Cell. 69 (1), 119–128. 10.1016/0092-8674(92)90123-t 1555236

[B47] Evens A. M. Balasubramanian S. Vose J. M. Harb W. Gordon L. I. Langdon R. (2016). A phase I/ii multicenter, open-label study of the oral histone deacetylase inhibitor abexinostat in relapsed/refractory lymphoma. Clin. Cancer Res. 22 (5), 1059–1066. Epub 2015/10/21. 10.1158/1078-0432.CCR-15-0624 26482040

[B48] Feldman T. Mato A. R. Chow K. F. Protomastro E. A. Yannotti K. M. Bhattacharyya P. (2014). Addition of lenalidomide to rituximab, ifosfamide, carboplatin, etoposide (ricer) in first-relapse/primary refractory diffuse large B-cell lymphoma. Br. J. Haematol. 166 (1), 77–83. Epub 20140325. 10.1111/bjh.12846 24661044 PMC4283736

[B49] Ferreri A. J. Sassone M. Zaja F. Re A. Spina M. Rocco A. D. (2017). Lenalidomide maintenance in patients with relapsed diffuse large B-cell lymphoma who are not eligible for autologous stem cell transplantation: An open label, single-arm, multicentre phase 2 trial. Lancet Haematol. 4 (3), e137–e146. Epub 20170217. 10.1016/S2352-3026(17)30016-9 28219694

[B50] Feugier P. Van Hoof A. Sebban C. Solal-Celigny P. Bouabdallah R. Ferme C. (2005). Long-term results of the R-chop study in the treatment of elderly patients with diffuse large B-cell lymphoma: A study by the groupe D'etude des lymphomes de L'adulte. J. Clin. Oncol. 23 (18), 4117–4126. Epub 20050502. 10.1200/JCO.2005.09.131 15867204

[B51] Franco F. Gonzalez-Rincon J. Lavernia J. Garcia J. F. Martin P. Bellas C. (2017). Mutational profile of primary breast diffuse large B-cell lymphoma. Oncotarget 8 (61), 102888–102897. Epub 20171024. 10.18632/oncotarget.21986 29262531 PMC5732697

[B52] Friedberg J. W. (2011). Relapsed/refractory diffuse large B-cell lymphoma. Hematol. Am. Soc. Hematol. Educ. Program 2011 (11), 498–505. 10.1182/asheducation-2011.1.498 22160081

[B53] Friedberg J. W. Sharman J. Sweetenham J. Johnston P. B. Vose J. M. Lacasce A. (2010). Inhibition of syk with fostamatinib disodium has significant clinical activity in non-hodgkin lymphoma and chronic lymphocytic leukemia. Blood 115 (13), 2578–2585. Epub 20091117. 10.1182/blood-2009-08-236471 19965662 PMC2852362

[B54] Fukumura K. Kawazu M. Kojima S. Ueno T. Sai E. Soda M. (2016). Genomic characterization of primary central nervous system lymphoma. Acta Neuropathol. 131 (6), 865–875. Epub 20160112. 10.1007/s00401-016-1536-2 26757737

[B55] Gao J. Aksoy B. A. Dogrusoz U. Dresdner G. Gross B. Sumer S. O. (2013). Integrative analysis of complex cancer genomics and clinical profiles using the cbioportal. Sci. Signal 6 (269), pl1. Epub 20130402. 10.1126/scisignal.2004088 23550210 PMC4160307

[B56] Georgiou K. Chen L. Berglund M. Ren W. de Miranda N. F. Lisboa S. (2016). Genetic basis of Pd-L1 overexpression in diffuse large B-cell lymphomas. Blood 127 (24), 3026–3034. Epub 20160330. 10.1182/blood-2015-12-686550 27030389

[B57] Gisselbrecht C. Glass B. Mounier N. Singh Gill D. Linch D. C. Trneny M. (2010). Salvage regimens with autologous transplantation for relapsed large B-cell lymphoma in the rituximab era. J. Clin. Oncol. 28 (27), 4184–4190. Epub 20100726. 10.1200/JCO.2010.28.1618 20660832 PMC3664033

[B58] Gordon M. S. Kanegai C. M. Doerr J. R. Wall R. (2003). Somatic hypermutation of the B cell receptor genes B29 (igbeta, Cd79b) and Mb1 (igalpha, Cd79a). Proc. Natl. Acad. Sci. U. S. A. 100 (7), 4126–4131. Epub 20030321. 10.1073/pnas.0735266100 12651942 PMC153059

[B59] Goy A. Younes A. McLaughlin P. Pro B. Romaguera J. E. Hagemeister F. (2005). Phase ii study of proteasome inhibitor bortezomib in relapsed or refractory B-cell non-hodgkin's lymphoma. J. Clin. Oncol. 23 (4), 667–675. Epub 20041221. 10.1200/JCO.2005.03.108 15613697

[B60] Green J. A. Suzuki K. Cho B. Willison L. D. Palmer D. Allen C. D. (2011). The sphingosine 1-phosphate receptor S1P₂ maintains the homeostasis of germinal center B cells and promotes niche confinement. Nat. Immunol. 12 (7), 672–680. Epub 20110605. 10.1038/ni.2047 21642988 PMC3158008

[B61] Gu J. J. Thompson S. J. Mavis C. Barth M. J. Torka P. Hernandez-Ilizaliturri F. J. (2019). Targeting Mdm2 and xiap by idasanutlin in diffuse large B-cell lymphoma. Blood 134, 5301. 10.1182/blood-2019-129009

[B62] Hainsworth J. D. Arrowsmith E. R. McCleod M. Hsi E. D. Hamid O. Shi P. (2016). A randomized, phase 2 study of R-chop plus enzastaurin vs R-chop in patients with intermediate- or high-risk diffuse large B-cell lymphoma. Leuk. Lymphoma 57 (1), 216–218. Epub 20151005. 10.3109/10428194.2015.1045898 25956042

[B63] Hans C. P. Weisenburger D. D. Greiner T. C. Gascoyne R. D. Delabie J. Ott G. (2004). Confirmation of the molecular classification of diffuse large B-cell lymphoma by immunohistochemistry using a tissue microarray. Blood 103 (1), 275–282. Epub 20030922. 10.1182/blood-2003-05-1545 14504078

[B64] Hashwah H. Schmid C. A. Kasser S. Bertram K. Stelling A. Manz M. G. (2017). Inactivation of crebbp expands the germinal center B cell compartment, down-regulates mhcii expression and promotes dlbcl growth. Proc. Natl. Acad. Sci. U. S. A. 114 (36), 9701–9706. Epub 20170822. 10.1073/pnas.1619555114 28831000 PMC5594639

[B65] Hatzi K. Jiang Y. Huang C. Garrett-Bakelman F. Gearhart M. D. Giannopoulou E. G. (2013). A hybrid mechanism of action for Bcl6 in B cells defined by formation of functionally distinct complexes at enhancers and promoters. Cell. Rep. 4 (3), 578–588. Epub 20130801. 10.1016/j.celrep.2013.06.016 23911289 PMC3854650

[B66] Havranek O. Xu J. Kohrer S. Wang Z. Becker L. Comer J. M. (2017). Tonic B-cell receptor signaling in diffuse large B-cell lymphoma. Blood 130 (8), 995–1006. Epub 20170623. 10.1182/blood-2016-10-747303 28646116 PMC5813722

[B67] Hernandez-Ilizaliturri F. J. Deeb G. Zinzani P. L. Pileri S. A. Malik F. Macon W. R. (2011). Higher response to lenalidomide in relapsed/refractory diffuse large B-cell lymphoma in nongerminal center B-Cell-Like than in germinal center B-Cell-Like phenotype. Cancer 117 (22), 5058–5066. Epub 20110414. 10.1002/cncr.26135 21495023

[B68] Hitz F. Fischer N. Pabst T. Caspar C. Berthod G. Eckhardt K. (2013). Rituximab, bendamustine, and lenalidomide in patients with aggressive B cell lymphoma not eligible for high-dose chemotherapy or anthracycline-based therapy: Phase I results of the sakk 38/08 trial. Ann. Hematol. 92 (8), 1033–1040. Epub 20130417. 10.1007/s00277-013-1751-z 23592273

[B69] Hitz F. Zucca E. Pabst T. Fischer N. Cairoli A. Samaras P. (2016). Rituximab, bendamustine and lenalidomide in patients with aggressive B-cell lymphoma not eligible for anthracycline-based therapy or intensive salvage chemotherapy - sakk 38/08. Br. J. Haematol. 174 (2), 255–263. Epub 20160328. 10.1111/bjh.14049 27018242

[B70] Hummel M. Bentink S. Berger H. Klapper W. Wessendorf S. Barth T. F. (2006). A biologic definition of burkitt's lymphoma from transcriptional and genomic profiling. N. Engl. J. Med. 354 (23), 2419–2430. 10.1056/NEJMoa055351 16760442

[B71] Intlekofer A. M. Younes A. (2014). Precision therapy for lymphoma--current state and future directions. Nat. Rev. Clin. Oncol. 11 (10), 585–596. Epub 20140819. 10.1038/nrclinonc.2014.137 25135367

[B72] Iqbal J. Sanger W. G. Horsman D. E. Rosenwald A. Pickering D. L. Dave B. (2004). Bcl2 translocation defines a unique tumor subset within the germinal center B-Cell-Like diffuse large B-cell lymphoma. Am. J. Pathol. 165 (1), 159–166. 10.1016/s0002-9440(10)63284-1 15215171 PMC1618550

[B73] Italiano A. Soria J. C. Toulmonde M. Michot J. M. Lucchesi C. Varga A. (2018). Tazemetostat, an Ezh2 inhibitor, in relapsed or refractory B-cell non-hodgkin lymphoma and advanced solid Tumours: A first-in-human, open-label, phase 1 study. Lancet Oncol. 19 (5), 649–659. Epub 20180409. 10.1016/S1470-2045(18)30145-1 29650362

[B74] Iyer G. Hanrahan A. J. Milowsky M. I. Al-Ahmadie H. Scott S. N. Janakiraman M. (2012). Genome sequencing identifies a basis for everolimus sensitivity. Science 338 (6104), 221. Epub 20120823. 10.1126/science.1226344 22923433 PMC3633467

[B75] Jardin F. Jais J. P. Molina T. J. Parmentier F. Picquenot J. M. Ruminy P. (2010). Diffuse large B-cell lymphomas with Cdkn2a deletion have a distinct gene expression signature and a poor prognosis under R-chop treatment: A gela study. Blood 116 (7), 1092–1104. Epub 20100430. 10.1182/blood-2009-10-247122 20435884

[B76] Jia Z. He J. Cen L. Han W. Jiang N. Yang J. (2012). P53 deletion is independently associated with increased age and decreased survival in a cohort of Chinese patients with diffuse large B-cell lymphoma. Leuk. Lymphoma 53 (11), 2182–2185. Epub 20120521. 10.3109/10428194.2012.686106 22545859

[B77] Jiang Y. Melnick A. (2015). The epigenetic basis of diffuse large B-cell lymphoma. Semin. Hematol. 52 (2), 86–96. Epub 20150119. 10.1053/j.seminhematol.2015.01.003 25805588 PMC4374125

[B78] Jiang Y. Ortega-Molina A. Geng H. Ying H. Y. Hatzi K. Parsa S. (2017). Crebbp inactivation promotes the development of hdac3-dependent lymphomas. Cancer Discov. 7 (1), 38–53. Epub 20161012. 10.1158/2159-8290.CD-16-0975 27733359 PMC5300005

[B79] Johnston P. B. LaPlant B. McPhail E. Habermann T. M. Inwards D. J. Micallef I. N. (2016). Everolimus combined with R-chop-21 for new, untreated, diffuse large B-cell lymphoma (ncctg 1085 [alliance]): Safety and efficacy results of a phase 1 and feasibility trial. Lancet Haematol. 3 (7), e309–e316. Epub 20160605. 10.1016/S2352-3026(16)30040-0 27374464 PMC4958393

[B80] Justin Kline M. D. J. G. M. Tumuluru Sravya Girish Venkataraman M. D. M. B. B. S. Robert J. Orlowski M. D. Sonali M. (2018). Pd-L1 gene alterations identify a unique subset of diffuse large B cell lymphoma that harbors a T cell inflamed phenotype. Blood 132, 673. 10.1182/blood-2018-99-117189 30910787 PMC6911840

[B81] Kaelin W. G. Jr. (2005). The concept of synthetic lethality in the context of anticancer therapy. Nat. Rev. Cancer 5 (9), 689–698. 10.1038/nrc1691 16110319

[B82] Karube K. Campo E. (2015). Myc alterations in diffuse large B-cell lymphomas. Semin. Hematol. 52 (2), 97–106. Epub 20150121. 10.1053/j.seminhematol.2015.01.009 25805589

[B83] Kent W. J. (2002). Blat--the blast-like alignment tool. Genome Res. 12 (4), 656–664. 10.1101/gr.229202 11932250 PMC187518

[B84] Kraan W. van Keimpema M. Horlings H. M. Schilder-Tol E. J. Oud M. E. Noorduyn L. A. (2014). High prevalence of oncogenic Myd88 and Cd79b mutations in primary testicular diffuse large B-cell lymphoma. Leukemia 28 (3), 719–720. Epub 20131120. 10.1038/leu.2013.348 24253023

[B85] Kridel R. Sehn L. H. Gascoyne R. D. (2012). Pathogenesis of follicular lymphoma. J. Clin. Investig. 122 (10), 3424–3431. Epub 20121001. 10.1172/JCI63186 23023713 PMC3461914

[B86] Kurland J. F. Tansey W. P. (2008). Myc-mediated transcriptional repression by recruitment of histone deacetylase. Cancer Res. 68 (10), 3624–3629. 10.1158/0008-5472.CAN-07-6552 18483244

[B87] Kusumoto S. Kobayashi Y. Sekiguchi N. Tanimoto K. Onishi Y. Yokota Y. (2005). Diffuse large B-cell lymphoma with extra bcl-2 gene signals detected by fish analysis is associated with a "Non-Germinal center phenotype. Am. J. Surg. Pathol. 29 (8), 1067–1073. 10.1097/01.pas.0000167362.06148.95 16006802

[B88] Lacy S. E. Barrans S. L. Beer P. A. Painter D. Smith A. G. Roman E. (2020). Targeted sequencing in dlbcl, molecular subtypes, and outcomes: A haematological malignancy research Network report. Blood 135 (20), 1759–1771. 10.1182/blood.2019003535 32187361 PMC7259825

[B89] Lai C. Roschewski M. Melani C. Pittaluga S. Shovlin M. Steinberg S. M. (2018). Myc gene rearrangement in diffuse large B-cell lymphoma does not confer a worse prognosis following dose-adjusted epoch-R. Leuk. Lymphoma 59 (2), 505–508. Epub 20170622. 10.1080/10428194.2017.1339882 28641474 PMC6368822

[B90] Lam L. T. Wright G. Davis R. E. Lenz G. Farinha P. Dang L. (2008). Cooperative signaling through the signal transducer and activator of transcription 3 and nuclear Factor-{Kappa}B pathways in subtypes of diffuse large B-cell lymphoma. Blood 111 (7), 3701–3713. Epub 20071226. 10.1182/blood-2007-09-111948 18160665 PMC2275028

[B91] Lamason R. L. McCully R. R. Lew S. M. Pomerantz J. L. (2010). Oncogenic Card11 mutations induce hyperactive signaling by disrupting autoinhibition by the pkc-responsive inhibitory domain. Biochemistry 49 (38), 8240–8250. 10.1021/bi101052d 20799731 PMC2943563

[B92] Lee S. Y. Kumano K. Nakazaki K. Sanada M. Matsumoto A. Yamamoto G. (2009). Gain-of-Function mutations and copy number increases of Notch2 in diffuse large B-cell lymphoma. Cancer Sci. 100 (5), 920–926. 10.1111/j.1349-7006.2009.01130.x 19445024 PMC11158873

[B93] Lenz G. Davis R. E. Ngo V. N. Lam L. George T. C. Wright G. W. (2008). Oncogenic Card11 mutations in human diffuse large B cell lymphoma. Science 319 (5870), 1676–1679. Epub 20080306. 10.1126/science.1153629 18323416

[B94] Lenz G. Nagel I. Siebert R. Roschke A. V. Sanger W. Wright G. W. (2007). Aberrant immunoglobulin class switch recombination and switch translocations in activated B cell-like diffuse large B cell lymphoma. J. Exp. Med. 204 (3), 633–643. Epub 2007/03/14. 10.1084/jem.20062041 17353367 PMC2137913

[B95] Lenz G. Wright G. W. Emre N. C. Kohlhammer H. Dave S. S. Davis R. E. (2008). Molecular subtypes of diffuse large B-cell lymphoma arise by distinct genetic pathways. Proc. Natl. Acad. Sci. U. S. A. 105 (36), 13520–13525. Epub 20080902. 10.1073/pnas.0804295105 18765795 PMC2533222

[B96] Leonard J. P. Kolibaba K. S. Reeves J. A. Tulpule A. Flinn I. W. Kolevska T. (2017). Randomized phase ii study of R-chop with or without bortezomib in previously untreated patients with non-germinal center B-Cell-Like diffuse large B-cell lymphoma. J. Clin. Oncol. 35 (31), 3538–3546. Epub 20170901. 10.1200/JCO.2017.73.2784 28862883

[B97] Lesokhin A. M. Ansell S. M. Armand P. Scott E. C. Halwani A. Gutierrez M. (2016). Nivolumab in patients with relapsed or refractory hematologic malignancy: Preliminary results of a phase ib study. J. Clin. Oncol. 34 (23), 2698–2704. Epub 20160606. 10.1200/JCO.2015.65.9789 27269947 PMC5019749

[B98] Li H. Durbin R. (2009). Fast and accurate short read alignment with burrows-wheeler transform. Bioinformatics 25 (14), 1754–1760. Epub 20090518. 10.1093/bioinformatics/btp324 19451168 PMC2705234

[B99] Li W. Gupta S. K. Han W. Kundson R. A. Nelson S. Knutson D. (2019). Targeting myc activity in double-hit lymphoma with myc and Bcl2 and/or Bcl6 rearrangements with epigenetic bromodomain inhibitors. J. Hematol. Oncol. 12 (1), 73. Epub 20190709. 10.1186/s13045-019-0761-2 31288832 PMC6617630

[B100] Lieber M. R. (2016). Mechanisms of human lymphoid chromosomal translocations. Nat. Rev. Cancer 16 (6), 387–398. Epub 2016/05/26. 10.1038/nrc.2016.40 27220482 PMC5336345

[B101] Liu Y. Mondello P. Erazo T. Tannan N. B. Asgari Z. de Stanchina E. (2018). Noxa genetic amplification or pharmacologic induction primes lymphoma cells to Bcl2 inhibitor-induced cell death. Proc. Natl. Acad. Sci. U. S. A. 115 (47), 12034–12039. Epub 20181107. 10.1073/pnas.1806928115 30404918 PMC6255185

[B102] Lohr J. G. Stojanov P. Lawrence M. S. Auclair D. Chapuy B. Sougnez C. (2012). Discovery and prioritization of somatic mutations in diffuse large B-cell lymphoma (dlbcl) by whole-exome sequencing. Proc. Natl. Acad. Sci. U. S. A. 109 (10), 3879–3884. Epub 20120217. 10.1073/pnas.1121343109 22343534 PMC3309757

[B103] Lu W. Ning H. Gu L. Peng H. Wang Q. Hou R. (2016). Mcpip1 selectively destabilizes transcripts associated with an antiapoptotic gene expression program in breast cancer cells that can elicit complete tumor regression. Cancer Res. 76 (6), 1429–1440. Epub 20160201. 10.1158/0008-5472.CAN-15-1115 26833120 PMC4794406

[B104] Ma A. Malynn B. A. (2020). A20: Linking a complex regulator of ubiquitylation to immunity and human disease. Nat. Rev. Immunol. 12 (11), 774–785. Epub 20121012. 10.1038/nri3313 PMC3582397 23059429

[B105] Major A. Kline J. Karrison T. G. Fishkin P. A. S. Kimball A. S. Petrich A. M. (2022). Phase I/ii clinical trial of temsirolimus and lenalidomide in patients with relapsed and refractory lymphomas. Haematologica 107 (7), 1608–1618. Epub 2021/07/30. 10.3324/haematol.2021.278853 34320785 PMC9244831

[B106] Mandelbaum J. Bhagat G. Tang H. Mo T. Brahmachary M. Shen Q. (2010). Blimp1 is a tumor suppressor gene frequently disrupted in activated B cell-like diffuse large B cell lymphoma. Cancer Cell. 18 (6), 568–579. 10.1016/j.ccr.2010.10.030 21156281 PMC3030476

[B107] Mansouri L. Thorvaldsdottir B. Laidou S. Stamatopoulos K. Rosenquist R. (2022). Precision diagnostics in lymphomas - recent developments and future directions. Semin. Cancer Biol. 84, 170–183. Epub 20211023. 10.1016/j.semcancer.2021.10.007 34699973

[B108] Martin A. Redondo A. M. Dlouhy I. Salar A. Gonzalez-Barca E. Canales M. (2016). Lenalidomide in combination with R-eshap in patients with relapsed or refractory diffuse large B-cell lymphoma: A phase 1b study from geltamo group. Br. J. Haematol. 173 (2), 245–252. Epub 20160205. 10.1111/bjh.13945 26847165

[B109] Miao Y. Medeiros L. J. Li Y. Li J. Young K. H. (2019). Genetic alterations and their clinical implications in dlbcl. Nat. Rev. Clin. Oncol. 16 (10), 634–652. 10.1038/s41571-019-0225-1 31127191

[B110] Milowsky M. I. Iyer G. Regazzi A. M. Al-Ahmadie H. Gerst S. R. Ostrovnaya I. (2013). Phase ii study of everolimus in metastatic urothelial cancer. BJU Int. 112 (4), 462–470. Epub 20130403. 10.1111/j.1464-410X.2012.11720.x 23551593 PMC4020005

[B111] Mondello P. Derenzini E. Asgari Z. Philip J. Brea E. J. Seshan V. (2017). Dual inhibition of histone deacetylases and phosphoinositide 3-kinase enhances therapeutic activity against B cell lymphoma. Oncotarget 8 (8), 14017–14028. 10.18632/oncotarget.14876 28147336 PMC5355158

[B112] Montesinos-Rongen M. Godlewska E. Brunn A. Wiestler O. D. Siebert R. Deckert M. (2011). Activating L265p mutations of the Myd88 gene are common in primary central nervous system lymphoma. Acta Neuropathol. 122 (6), 791–792. Epub 20111022. 10.1007/s00401-011-0891-2 22020631

[B113] Monti S. Savage K. J. Kutok J. L. Feuerhake F. Kurtin P. Mihm M. (2005). Molecular profiling of diffuse large B-cell lymphoma identifies robust subtypes including one characterized by host inflammatory response. Blood 105 (5), 1851–1861. Epub 20041118. 10.1182/blood-2004-07-2947 15550490

[B114] Morin R. D. Mendez-Lago M. Mungall A. J. Goya R. Mungall K. L. Corbett R. D. (2011). Frequent mutation of histone-modifying genes in non-hodgkin lymphoma. Nature 476 (7360), 298–303. Epub 20110727. 10.1038/nature10351 21796119 PMC3210554

[B115] Morschhauser F. Feugier P. Flinn I. W. Gasiorowski R. Greil R. Illes A. (2021). A phase 2 study of venetoclax plus R-CHOP as first-line treatment for patients with diffuse large B-cell lymphoma. Blood 137 (5), 600–609. 10.1182/blood.2020006578 33538797 PMC7869186

[B116] Mottok A. Gascoyne R. D. (2015). Bromodomain inhibition in diffuse large B-cell lymphoma--giving myc a brake. Clin. Cancer Res. 21 (1), 4–6. Epub 20140827. 10.1158/1078-0432.CCR-14-1651 25165099

[B117] Muppidi J. R. Schmitz R. Green J. A. Xiao W. Larsen A. B. Braun S. E. (2014). Loss of signalling via Gα13 in germinal centre B-cell-derived lymphoma. Nature 516 (7530), 254–258. Epub 20140928. 10.1038/nature13765 25274307 PMC4267955

[B118] Muramatsu M. Kinoshita K. Fagarasan S. Yamada S. Shinkai Y. Honjo T. (2000). Class switch recombination and hypermutation require activation-induced cytidine deaminase (aid), a potential rna editing enzyme. Cell. 102 (5), 553–563. Epub 2000/09/28. 10.1016/s0092-8674(00)00078-7 11007474

[B119] Nayak L. Iwamoto F. M. LaCasce A. Mukundan S. Roemer M. G. M. Chapuy B. (2017). Pd-1 blockade with nivolumab in relapsed/refractory primary central nervous system and testicular lymphoma. Blood 129 (23), 3071–3073. Epub 20170329. 10.1182/blood-2017-01-764209 28356247 PMC5766844

[B120] NCI Genomic Data Commons (2016). NCI Genomic Data Commons. Available at: https://gdc.cancer.gov (Accessed October 22, 2022).

[B121] Ngo V. N. Young R. M. Schmitz R. Jhavar S. Xiao W. Lim K. H. (2011). Oncogenically active Myd88 mutations in human lymphoma. Nature 470 (7332), 115–119. Epub 20101222. 10.1038/nature09671 21179087 PMC5024568

[B35] Nicorici D. Şatalan M. Edgren H. Kangaspeska S. Murumägi A. Kallioniemi O. (2014). FusionCatcher – a tool for finding somatic fusion genes in paired-end RNA-sequencing data. bioRxiv. 10.1101/011650

[B122] Notch1 Gene MedlinePlus. (2015). NOTCH1 gene: MedlinePlus Genetics. Available from: https://medlineplus.gov/genetics/gene/notch1/#conditions (Accessed October 22, 2022).

[B123] Nowakowski G. S. LaPlant B. Macon W. R. Reeder C. B. Foran J. M. Nelson G. D. (2015). Lenalidomide combined with R-chop overcomes negative prognostic impact of non-germinal center B-cell phenotype in newly diagnosed diffuse large B-cell lymphoma: A phase ii study. J. Clin. Oncol. 33 (3), 251–257. Epub 20140818. 10.1200/JCO.2014.55.5714 25135992

[B124] Offner F. Samoilova O. Osmanov E. Eom H. S. Topp M. S. Raposo J. (2015). Frontline rituximab, cyclophosphamide, doxorubicin, and prednisone with bortezomib (Vr-Cap) or vincristine (R-Chop) for non-gcb dlbcl. Blood 126 (16), 1893–1901. Epub 20150731. 10.1182/blood-2015-03-632430 26232170 PMC4616024

[B125] Oishi N. Kondo T. Nakazawa T. Mochizuki K. Tanioka F. Oyama T. (2015). High prevalence of the Myd88 mutation in testicular lymphoma: Immunohistochemical and genetic analyses. Pathol. Int. 65 (10), 528–535. Epub 20150804. 10.1111/pin.12336 26388135

[B126] Oki Y. Fanale M. Romaguera J. Fayad L. Fowler N. Copeland A. (2015). Phase ii study of an akt inhibitor Mk2206 in patients with relapsed or refractory lymphoma. Br. J. Haematol. 171 (4), 463–470. Epub 20150727. 10.1111/bjh.13603 26213141 PMC5278973

[B127] Oki Y. Kelly K. R. Flinn I. Patel M. R. Gharavi R. Ma A. (2017). Cudc-907 in relapsed/refractory diffuse large B-cell lymphoma, including patients with myc-alterations: Results from an expanded phase I trial. Haematologica 102 (11), 1923–1930. Epub 20170831. 10.3324/haematol.2017.172882 28860342 PMC5664396

[B128] Ortega-Molina A. Boss I. W. Canela A. Pan H. Jiang Y. Zhao C. (2015). The histone lysine methyltransferase Kmt2d sustains a gene expression program that represses B cell lymphoma development. Nat. Med. 21 (10), 1199–1208. Epub 20150914. 10.1038/nm.3943 26366710 PMC4676270

[B129] Papageorgiou S. G. Thomopoulos T. P. Katagas I. Bouchla A. Pappa V. (2021). Prognostic molecular biomarkers in diffuse large B-cell lymphoma in the rituximab era and their therapeutic implications. Ther. Adv. Hematol. 12, 20406207211013987. Epub 20210524. 10.1177/20406207211013987 34104369 PMC8150462

[B130] Pasqualucci L. Compagno M. Houldsworth J. Monti S. Grunn A. Nandula S. V. (2006). Inactivation of the prdm1/blimp1 gene in diffuse large B cell lymphoma. J. Exp. Med. 203 (2), 311–317. 10.1084/jem.20052204 16492805 PMC2118216

[B131] Pasqualucci L. Dominguez-Sola D. Chiarenza A. Fabbri G. Grunn A. Trifonov V. (2011). Inactivating mutations of acetyltransferase genes in B-cell lymphoma. Nature 471 (7337), 189–195. 10.1038/nature09730 21390126 PMC3271441

[B132] Pasqualucci L. Trifonov V. Fabbri G. Ma J. Rossi D. Chiarenza A. (2011). Analysis of the coding genome of diffuse large B-cell lymphoma. Nat. Genet. 43 (9), 830–837. Epub 20110731. 10.1038/ng.892 21804550 PMC3297422

[B133] Paul J. Soujon M. Wengner A. M. Zitzmann-Kolbe S. Sturz A. Haike K. (2017). Simultaneous inhibition of PI3Kδ and PI3Kα induces ABC-DLBCL regression by blocking BCR-dependent and -independent activation of NF-κB and AKT. Cancer Cell. 31 (1), 64–78. Epub 2017/01/11. 10.1016/j.ccell.2016.12.003 28073005

[B134] Pfeifer M. Grau M. Lenze D. Wenzel S. S. Wolf A. Wollert-Wulf B. (2013). Pten loss defines a pi3k/akt pathway-dependent germinal center subtype of diffuse large B-cell lymphoma. Proc. Natl. Acad. Sci. U. S. A. 110 (30), 12420–12425. Epub 20130709. 10.1073/pnas.1305656110 23840064 PMC3725065

[B135] Pfreundschuh M. Schubert J. Ziepert M. Schmits R. Mohren M. Lengfelder E. (2008). Six versus eight cycles of Bi-weekly chop-14 with or without rituximab in elderly patients with aggressive Cd20+ B-cell lymphomas: A randomised controlled trial (Ricover-60). Lancet Oncol. 9 (2), 105–116. Epub 20080115. 10.1016/S1470-2045(08)70002-0 18226581

[B136] Pfreundschuh M. Trumper L. Osterborg A. Pettengell R. Trneny M. Imrie K. (2006). Chop-like chemotherapy plus rituximab versus chop-like chemotherapy alone in Young patients with good-prognosis diffuse large-B-cell lymphoma: A randomised controlled trial by the mabthera international trial (mint) group. Lancet Oncol. 7 (5), 379–391. 10.1016/S1470-2045(06)70664-7 16648042

[B137] Pham-Ledard A. Beylot-Barry M. Barbe C. Leduc M. Petrella T. Vergier B. (2014). High frequency and clinical prognostic value of Myd88 L265p mutation in primary cutaneous diffuse large B-cell lymphoma, leg-type. JAMA Dermatol 150 (11), 1173–1179. 10.1001/jamadermatol.2014.821 25055137

[B138] Pham-Ledard A. Cappellen D. Martinez F. Vergier B. Beylot-Barry M. Merlio J. P. (2012). Myd88 somatic mutation is a genetic feature of primary cutaneous diffuse large B-cell lymphoma, leg type. J. Investig. Dermatol 132 (8), 2118–2120. Epub 20120412. 10.1038/jid.2012.102 22495176

[B139] Pham-Ledard A. Prochazkova-Carlotti M. Andrique L. Cappellen D. Vergier B. Martinez F. (2014). Multiple genetic alterations in primary cutaneous large B-cell lymphoma, leg type support a common lymphomagenesis with activated B-Cell-Like diffuse large B-cell lymphoma. Mod. Pathol. 27 (3), 402–411. Epub 20130913. 10.1038/modpathol.2013.156 24030746

[B140] Phelan J. D. Young R. M. Webster D. E. Roulland S. Wright G. W. Kasbekar M. (2018). A multiprotein supercomplex controlling oncogenic signalling in lymphoma. Nature 560 (7718), 387–391. Epub 20180620. 10.1038/s41586-018-0290-0 29925955 PMC6201842

[B141] Puente X. S. Bea S. Valdes-Mas R. Villamor N. Gutierrez-Abril J. Martin-Subero J. I. (2015). Non-coding recurrent mutations in chronic lymphocytic leukaemia. Nature 526 (7574), 519–524. Epub 20150722. 10.1038/nature14666 26200345

[B142] Radford John A. McKay Pamela Cartron Guillaume Pimentel Patricia J. Roche Maria Blakemore Stephen J. (2016). Phase 2 multi-center study of tazemetostat (Epz-6438), an inhibitor of enhancer of zeste-homolog 2 (Ezh2), in patients with relapsed or refractory B-cell non-hodgkin lymphoma (nhl). J. Clin. Oncol. 34 (15), TPS7582. 10.1200/jco.2016.34.15_suppl.tps7582

[B143] Rawlings D. J. Metzler G. Wray-Dutra M. Jackson S. W. (2017). Altered B cell signalling in autoimmunity. Nat. Rev. Immunol. 17 (7), 421–436. Epub 20170410. 10.1038/nri.2017.24 28393923 PMC5523822

[B144] Reddy N. M. Greer J. P. Morgan D. S. Chen H. Park S. I. Richards K. L. (2017). A phase ii randomized study of lenalidomide or lenalidomide and rituximab as maintenance therapy following standard chemotherapy for patients with high/high-intermediate risk diffuse large B-cell lymphoma. Leukemia 31 (1), 241–244. Epub 20160922. 10.1038/leu.2016.255 27654851 PMC5214342

[B145] Robbiani D. F. Nussenzweig M. C. (2013). Chromosome translocation, B cell lymphoma, and activation-induced cytidine deaminase. Annu. Rev. Pathol. 8, 79–103. Epub 2012/09/15. 10.1146/annurev-pathol-020712-164004 22974238

[B146] Robertson M. J. Kahl B. S. Vose J. M. de Vos S. Laughlin M. Flynn P. J. (2007). Phase ii study of enzastaurin, a protein kinase C beta inhibitor, in patients with relapsed or refractory diffuse large B-cell lymphoma. J. Clin. Oncol. 25 (13), 1741–1746. Epub 20070326. 10.1200/JCO.2006.09.3146 17389337

[B147] Rosenquist R. Bea S. Du M. Q. Nadel B. Pan-Hammarstrom Q. (2017). Genetic Landscape and deregulated pathways in B-cell lymphoid malignancies. J. Intern Med. 282 (5), 371–394. Epub 20170620. 10.1111/joim.12633 28631441

[B148] Rosenquist R. Rosenwald A. Du M. Q. Gaidano G. Groenen P. Wotherspoon A. (2016). Clinical impact of recurrently mutated genes on lymphoma diagnostics: State-of-the-Art and beyond. Haematologica 101 (9), 1002–1009. 10.3324/haematol.2015.134510 27582569 PMC5060016

[B149] Sarkozy C. Morschhauser F. Dubois S. Molina T. Michot J. M. Cullieres-Dartigues P. (2020). A lysa phase ib study of tazemetostat (Epz-6438) plus R-chop in patients with newly diagnosed diffuse large B-cell lymphoma (dlbcl) with poor prognosis features. Clin. Cancer Res. 26 (13), 3145–3153. Epub 20200302. 10.1158/1078-0432.CCR-19-3741 32122924

[B150] Savage K. J. Johnson N. A. Ben-Neriah S. Connors J. M. Sehn L. H. Farinha P. (2009). Myc gene rearrangements are associated with a poor prognosis in diffuse large B-cell lymphoma patients treated with R-chop chemotherapy. Blood 114 (17), 3533–3537. Epub 20090824. 10.1182/blood-2009-05-220095 19704118

[B151] Schmitz R. Wright G. W. Huang D. W. Johnson C. A. Phelan J. D. Wang J. Q. (2018). Genetics and pathogenesis of diffuse large B-cell lymphoma. N. Engl. J. Med. 378 (15), 1396–1407. 10.1056/NEJMoa1801445 29641966 PMC6010183

[B152] Schuetz J. M. Johnson N. A. Morin R. D. Scott D. W. Tan K. Ben-Nierah S. (2012). Bcl2 mutations in diffuse large B-cell lymphoma. Leukemia 26 (6), 1383–1390. Epub 20111222. 10.1038/leu.2011.378 22189900

[B153] Scott D. W. Wright G. W. Williams P. M. Lih C. J. Walsh W. Jaffe E. S. (2014). Determining cell-of-origin subtypes of diffuse large B-cell lymphoma using gene expression in formalin-fixed paraffin-embedded tissue. Blood 123 (8), 1214–1217. Epub 20140107. 10.1182/blood-2013-11-536433 24398326 PMC3931191

[B154] Seshan V. E. O. A. (2017). Dnacopy: DNA copy number data analysis. R package version 1501.

[B155] Shaffer A. L. Wright G. Yang L. Powell J. Ngo V. Lamy L. (2006). A library of gene expression signatures to illuminate normal and pathological lymphoid biology. Immunol. Rev. 210, 67–85. 10.1111/j.0105-2896.2006.00373.x 16623765

[B156] Sharman J. S. A. Smith M. (2016). Updated results on the clinical activity of entospletinib (Gs-9973), a selective syk inhibitor, in patients with cll previously treated with an inhibitor of the B-cell receptor signaling pathway [abstract]. Blood (3225), 128.27827828

[B157] Shen H. M. Peters A. Baron B. Zhu X. Storb U. (1998). Mutation of bcl-6 gene in normal B cells by the process of somatic hypermutation of ig genes. Science 280 (5370), 1750–1752. 10.1126/science.280.5370.1750 9624052

[B158] Siddiqi T. Frankel P. Beumer J. H. Kiesel B. F. Christner S. Ruel C. (2020). Phase 1 study of the aurora kinase a inhibitor alisertib (Mln8237) combined with the histone deacetylase inhibitor vorinostat in lymphoid malignancies. Leuk. Lymphoma 61 (2), 309–317. Epub 2019/10/17. 10.1080/10428194.2019.1672052 31617432 PMC6982547

[B159] Skalniak L. Mizgalska D. Zarebski A. Wyrzykowska P. Koj A. Jura J. (2009). Regulatory feedback loop between nf-kappab and mcp-1-induced protein 1 rnase. FEBS J. 276 (20), 5892–5905. Epub 20090911. 10.1111/j.1742-4658.2009.07273.x 19747262

[B160] Staiger A. M. Ziepert M. Horn H. Scott D. W. Barth T. F. E. Bernd H. W. (2017). Clinical impact of the cell-of-origin classification and the myc/Bcl2 dual expresser status in diffuse large B-cell lymphoma treated within prospective clinical trials of the German high-grade non-hodgkin's lymphoma study group. J. Clin. Oncol. 35 (22), 2515–2526. Epub 20170519. 10.1200/JCO.2016.70.3660 28525305

[B161] Steimle V. Siegrist C. A. Mottet A. Lisowska-Grospierre B. Mach B. (1994). Regulation of mhc class ii expression by interferon-gamma mediated by the transactivator gene ciita. Science 265 (5168), 106–109. 10.1126/science.8016643 8016643

[B162] Stein H. Warnke R. A. Chan W. C. Jaffe E. S. Chan J. K. C. Gatter K. C. (2008). “Diffuse large B-cell lymphoma, not otherwise specified,” in WHO classification of Tumours of haematopoietic and lymphoid tissues. 4th Edition, 233–237.

[B163] Stewart C. M. Michaud L. Whiting K. Nakajima R. Nichols C. De Frank S. (2022). Phase I/ib study of the efficacy and safety of buparlisib and ibrutinib therapy in mcl, fl, and dlbcl with serial cell-free DNA monitoring. Clin. Cancer Res. 28 (1), 45–56. Epub 2021/10/08. 10.1158/1078-0432.CCR-21-2183 34615723 PMC8812724

[B164] Taniguchi K. Takata K. Chuang S. S. Miyata-Takata T. Sato Y. Satou A. (2016). Frequent Myd88 L265p and Cd79b mutations in primary breast diffuse large B-cell lymphoma. Am. J. Surg. Pathol. 40 (3), 324–334. 10.1097/PAS.0000000000000592 26752547

[B165] Thieblemont C. Tilly H. Gomes da Silva M. Casasnovas R. O. Fruchart C. Morschhauser F. (2017). Lenalidomide maintenance compared with placebo in responding elderly patients with diffuse large B-cell lymphoma treated with first-line rituximab plus cyclophosphamide, doxorubicin, vincristine, and prednisone. J. Clin. Oncol. 35 (22), 2473–2481. Epub 20170420. 10.1200/JCO.2017.72.6984 28426350

[B166] Tian T. Li X. Zhang J. (2019). Mtor signaling in cancer and mtor inhibitors in solid tumor targeting therapy. Int. J. Mol. Sci. 20 (3), 755. Epub 20190211. 10.3390/ijms20030755 30754640 PMC6387042

[B167] Tilly H. Gomes da Silva M. Vitolo U. Jack A. Meignan M. Lopez-Guillermo A. (2015). Diffuse large B-cell lymphoma (dlbcl): Esmo clinical practice guidelines for diagnosis, treatment and follow-up. Ann. Oncol. 26 (5), v116–v125. 10.1093/annonc/mdv304 26314773

[B168] Uddin S. Hussain A. R. Siraj A. K. Manogaran P. S. Al-Jomah N. A. Moorji A. (2006). Role of phosphatidylinositol 3'-kinase/akt pathway in diffuse large B-cell lymphoma survival. Blood 108 (13), 4178–4186. Epub 20060831. 10.1182/blood-2006-04-016907 16946303

[B169] Valera A. Lopez-Guillermo A. Cardesa-Salzmann T. Climent F. Gonzalez-Barca E. Mercadal S. (2013). Myc protein expression and genetic alterations have prognostic impact in patients with diffuse large B-cell lymphoma treated with immunochemotherapy. Haematologica 98 (10), 1554–1562. Epub 20130528. 10.3324/haematol.2013.086173 23716551 PMC3789460

[B170] Valls E. Lobry C. Geng H. Wang L. Cardenas M. Rivas M. (2017). Bcl6 antagonizes Notch2 to maintain survival of human follicular lymphoma cells. Cancer Discov. 7 (5), 506–521. Epub 20170223. 10.1158/2159-8290.CD-16-1189 28232365 PMC5413414

[B171] Velichutina I. Shaknovich R. Geng H. Johnson N. A. Gascoyne R. D. Melnick A. M. (2010). Ezh2-Mediated epigenetic silencing in germinal center B cells contributes to proliferation and lymphomagenesis. Blood 116 (24), 5247–5255. Epub 20100824. 10.1182/blood-2010-04-280149 20736451 PMC3012542

[B172] Vincent Ribrag F. M. McKay Pamela Gilles Andre Salles M. D. Connie Lee Batlevi M. D. Schmitt Anna Herve Tilly M. D. (2018). Interim results from an ongoing phase 2 multicenter study of tazemetostat, an Ezh2 inhibitor, in patients with relapsed or refractory (R/R) diffuse large B-cell lymphoma (dlbcl). Blood, 132.29866817

[B173] Visco C. Tzankov A. Xu-Monette Z. Y. Miranda R. N. Tai Y. C. Li Y. (2013). Patients with diffuse large B-cell lymphoma of germinal center origin with Bcl2 translocations have poor outcome, irrespective of myc status: A report from an international dlbcl rituximab-chop consortium program study. Haematologica 98 (2), 255–263. Epub 20120828. 10.3324/haematol.2012.066209 22929980 PMC3561433

[B174] Wang M. Fowler N. Wagner-Bartak N. Feng L. Romaguera J. Neelapu S. S. (2013). Oral lenalidomide with rituximab in relapsed or refractory diffuse large cell, follicular and transformed lymphoma: A phase ii clinical trial. Leukemia 27 (9), 1902–1909. Epub 20130402. 10.1038/leu.2013.95 23545991

[B175] Wang X. Cao X. Sun R. Tang C. Tzankov A. Zhang J. (2018). Clinical significance of pten deletion, mutation, and loss of pten expression in de novo diffuse large B-cell lymphoma. Neoplasia 20 (6), 574–593. Epub 20180504. 10.1016/j.neo.2018.03.002 29734016 PMC5994742

[B176] Wiernik P. H. Lossos I. S. Tuscano J. M. Justice G. Vose J. M. Cole C. E. (2008). Lenalidomide monotherapy in relapsed or refractory aggressive non-hodgkin's lymphoma. J. Clin. Oncol. 26 (30), 4952–4957. Epub 20080707. 10.1200/JCO.2007.15.3429 18606983

[B177] Wilson W. H. G. J. Goy A. de Vos S. Kenkre V. P. Barr P. M. Blum K. A. (2012). The Bruton's tyrosine kinase (BTK) inhibitor, ibrutinib (PCI-32765), has preferential activity in the ABC subtype of relapsed/refractory de novo diffuse large B-cell lymphoma (DLBCL): Interim results of a multicenter, open-label, phase 2 study. Blood (ASH Annu. Meet. Abstr 120, 686. 10.1182/blood.v120.21.686.686

[B178] Wilson W. H. Young R. M. Schmitz R. Yang Y. Pittaluga S. Wright G. (2015). Targeting B cell receptor signaling with ibrutinib in diffuse large B cell lymphoma. Nat. Med. 21 (8), 922–926. Epub 20150720. 10.1038/nm.3884 26193343 PMC8372245

[B179] Witzens-Harig M. Viardot A. Keller U. Wosniok J. Deuster O. Klemmer J. (2021). The mtor inhibitor temsirolimus added to rituximab combined with dexamethasone, cytarabine, and cisplatinum (R-Dhap) for the treatment of patients with relapsed or refractory dlbcl - results from the phase-ii storm trial. Hemasphere 5 (10), e636. Epub 2021/10/01. 10.1097/HS9.0000000000000636 34589671 PMC8476051

[B180] Witzig T. E. LaPlant B. Habermann T. M. McPhail E. Inwards D. J. Micallef I. N. (2017). High rate of event-free survival at 24 Months with everolimus/rchop for untreated diffuse large B-cell lymphoma: Updated results from ncctg N1085 (alliance). Blood Cancer J. 7 (6), e576. Epub 20170623. 10.1038/bcj.2017.57 28649983 PMC5520404

[B181] Witzig T. E. Vose J. M. Zinzani P. L. Reeder C. B. Buckstein R. Polikoff J. A. (2011). An international phase ii trial of single-agent lenalidomide for relapsed or refractory aggressive B-cell non-hodgkin's lymphoma. Ann. Oncol. 22 (7), 1622–1627. Epub 20110112. 10.1093/annonc/mdq626 21228334

[B182] Wright G. Tan B. Rosenwald A. Hurt E. H. Wiestner A. Staudt L. M. (2003). A gene expression-based method to diagnose clinically distinct subgroups of diffuse large B cell lymphoma. Proc. Natl. Acad. Sci. U. S. A. 100 (17), 9991–9996. Epub 20030804. 10.1073/pnas.1732008100 12900505 PMC187912

[B183] Wright G. W. Huang D. W. Phelan J. D. Coulibaly Z. A. Roulland S. Young R. M. (2020). A probabilistic classification tool for genetic subtypes of diffuse large B cell lymphoma with therapeutic implications. Cancer Cell. 37 (4), 551–568. 10.1016/j.ccell.2020.03.015 32289277 PMC8459709

[B184] Xu X. Zhang L. Wang Y. Zhang Q. Zhang L. Sun B. (2013). Double-hit and triple-hit lymphomas arising from follicular lymphoma following acquisition of myc: Report of two cases and literature review. Int. J. Clin. Exp. Pathol. 6 (4), 788–794. Epub 20130315. 23573328 PMC3606871

[B185] Xu-Monette Z. Y. Deng Q. Manyam G. C. Tzankov A. Li L. Xia Y. (2016). Clinical and biologic significance of myc genetic mutations in de novo diffuse large B-cell lymphoma. Clin. Cancer Res. 22 (14), 3593–3605. Epub 20160229. 10.1158/1078-0432.CCR-15-2296 26927665 PMC4947447

[B186] Xu-Monette Z. Y. Wu L. Visco C. Tai Y. C. Tzankov A. Liu W. M. (2012). Mutational profile and prognostic significance of Tp53 in diffuse large B-cell lymphoma patients treated with R-chop: Report from an international dlbcl rituximab-chop consortium program study. Blood 120 (19), 3986–3996. Epub 20120905. 10.1182/blood-2012-05-433334 22955915 PMC3496956

[B187] Yang Y. Shaffer A. L. 3rd Emre N. C. Ceribelli M. Zhang M. Wright G. (2012). Exploiting synthetic lethality for the therapy of abc diffuse large B cell lymphoma. Cancer Cell. 21 (6), 723–737. 10.1016/j.ccr.2012.05.024 22698399 PMC4059833

[B188] Younes A. Sehn L. H. Johnson P. Zinzani P. L. Hong X. Zhu J. (2019). Randomized phase iii trial of ibrutinib and rituximab plus cyclophosphamide, doxorubicin, vincristine, and prednisone in non-germinal center B-cell diffuse large B-cell lymphoma. J. Clin. Oncol. 37 (15), 1285–1295. Epub 20190322. 10.1200/JCO.18.02403 30901302 PMC6553835

[B189] Young R. M. Shaffer A. L. 3rd Phelan J. D. Staudt L. M. (2015). B-cell receptor signaling in diffuse large B-cell lymphoma. Semin. Hematol. 52 (2), 77–85. Epub 20150115. 10.1053/j.seminhematol.2015.01.008 25805587 PMC4374122

[B190] Young R. M. Staudt L. M. (2013). Targeting pathological B cell receptor signalling in lymphoid malignancies. Nat. Rev. Drug Discov. 12 (3), 229–243. 10.1038/nrd3937 23449308 PMC7595252

[B191] Zang C. Eucker J. Liu H. Coordes A. Lenarz M. Possinger K. (2014). Inhibition of pan-class I phosphatidyl-inositol-3-kinase by nvp-bkm120 effectively blocks proliferation and induces cell death in diffuse large B-cell lymphoma. Leuk. Lymphoma 55 (2), 425–434. Epub 2013/06/01. 10.3109/10428194.2013.806800 23721513

[B192] Zhang J. Dominguez-Sola D. Hussein S. Lee J. E. Holmes A. B. Bansal M. (2015). Disruption of Kmt2d perturbs germinal center B cell development and promotes lymphomagenesis. Nat. Med. 21 (10), 1190–1198. Epub 20150914. 10.1038/nm.3940 26366712 PMC5145002

[B193] Zhang L. H. Kosek J. Wang M. Heise C. Schafer P. H. Chopra R. (2013). Lenalidomide efficacy in activated B-Cell-Like subtype diffuse large B-cell lymphoma is dependent upon Irf4 and cereblon expression. Br. J. Haematol. 160 (4), 487–502. Epub 20121218. 10.1111/bjh.12172 23252516

[B194] Zhang X. Zhao X. Fiskus W. Lin J. Lwin T. Rao R. (2012). Coordinated silencing of myc-mediated mir-29 by Hdac3 and Ezh2 as a therapeutic target of histone modification in aggressive B-cell lymphomas. Cancer Cell. 22 (4), 506–523. 10.1016/j.ccr.2012.09.003 23079660 PMC3973134

[B195] Zheng M. Perry A. M. Bierman P. Loberiza F. Jr. Nasr M. R. Szwajcer D. (2017). Frequency of Myd88 and Cd79b mutations, and mgmt methylation in primary central nervous system diffuse large B-cell lymphoma. Neuropathology 37 (6), 509–516. Epub 20170830. 10.1111/neup.12405 28856744

[B196] Zhou X. A. Louissaint A. Jr. Wenzel A. Yang J. Martinez-Escala M. E. Moy A. P. (2018). Genomic analyses identify recurrent alterations in immune evasion genes in diffuse large B-cell lymphoma, leg type. J. Investig. Dermatol 138 (11), 2365–2376. Epub 20180530. 10.1016/j.jid.2018.04.038 29857068 PMC6200585

[B197] Zinzani P. L. Pellegrini C. Gandolfi L. Stefoni V. Quirini F. Derenzini E. (2011). Combination of lenalidomide and rituximab in elderly patients with relapsed or refractory diffuse large B-cell lymphoma: A phase 2 trial. Clin. Lymphoma Myeloma Leuk. 11 (6), 462–466. Epub 20110504. 10.1016/j.clml.2011.02.001 21859554

